# Closed-loop trans-skull ultrasound hyperthermia leads to improved drug delivery from thermosensitive drugs and promotes changes in vascular transport dynamics in brain tumors

**DOI:** 10.7150/thno.54630

**Published:** 2021-05-24

**Authors:** Chulyong Kim, Yutong Guo, Anastasia Velalopoulou, Johannes Leisen, Anjan Motamarry, Krishna Ramajayam, Muna Aryal, Dieter Haemmerich, Costas D. Arvanitis

**Affiliations:** 1 School of Mechanical Engineering, Georgia Institute of Technology, Atlanta, USA.; 2 Department of Biomedical Engineering, Georgia Institute of Technology and Emory University, Atlanta, GA, USA.; 3 Department of Chemistry and Biochemistry, Georgia Institute of Technology, Atlanta, GA, USA.; 4 Department of Dermatology, Massachusetts General Hospital, Harvard Medical School, Boston, MA, USA.; 5 Department of Pediatrics, Medical University of South Carolina, Charleston, SC, USA.; 6 Department of Radiology, Stanford University, Stanford, CA, USA.

**Keywords:** focused ultrasound, hyperthermia, thermosensitive drugs, thermal stress, brain cancer

## Abstract

Effective drug delivery in brain tumors remains a major challenge in oncology. Although local hyperthermia and stimuli-responsive delivery systems, such as thermosensitive liposomes, represent promising strategies to locally enhance drug delivery in solid tumors and improve outcomes, their application in intracranial malignancies remains unexplored. We hypothesized that the combined abilities of closed-loop trans-skull Magnetic Resonance Imaging guided Focused Ultrasound (MRgFUS) hyperthermia with those of thermosensitive drugs can alleviate challenges in drug delivery and improve survival in gliomas.

**Methods:** To conduct our investigations, we first designed a closed loop MR-guided Focused Ultrasound (MRgFUS) system for localized trans-skull hyperthermia (ΔT < 0.5 °C) in rodents and established safety thresholds in healthy mice. To assess the abilities of the developed system and proposed therapeutic strategy for FUS-triggered chemotherapy release we employed thermosensitive liposomal Dox (TSL-Dox) and tested it in two different glioma tumor models (F98 in rats and GL261 in mice). To quantify Dox delivery and changes in the transvascular transport dynamics in the tumor microenvironment we combined fluorescent microscopy, dynamic contrast enhanced MRI (DCE-MRI), and physiologically based pharmacokinetic (PBPK) modeling. Lastly, to assess the therapeutic efficacy of the system and of the proposed therapeutic strategy we performed a survival study in the GL261 glioma bearing mice.

**Results:** The developed closed-loop trans-skull MRgFUS-hyperthermia system that operated at 1.7 MHz, a frequency that maximized the brain (FUS-focus) to skull temperature ratio in mice, was able to attain and maintain the desired focal temperature within a narrow range. Histological evidence (H&E and Nissl) suggests that focal temperature at 41.5 ± 0.5 °C for 10 min is below the threshold for tissue damage. Quantitative analysis of doxorubicin delivery from TSLs with MRgFUS-hyperthermia demonstrated 3.5-fold improvement in cellular uptake in GL261 glioma mouse tumors (p < 0.001) and 5-fold increase in delivery in F98 glioma rat tumors (p < 0.05), as compared to controls (TSL-Dox-only). Moreover, PBPK modeling of drug transport that was calibrated using the experimental data indicated that thermal stress could lead to significant improvement in the transvascular transport (2.3-fold increase in the vessel diffusion coefficient; P < 0.001), in addition to promoting targeted Dox release. Prospective experimental investigations with DCE-MRI during FUS-hyperthermia, supported these findings and provided evidence that moderate thermal stress (≈41 °C for up to 10 min) can promote acute changes in the vascular transport dynamics in the brain tumor microenvironment (K^trans^ value for control vs. FUS was 0.0097 and 0.0148 min^-1^, respectively; p = 0.026). Crucially, survival analysis demonstrated significant improvement in the survival in the TSL-Dox-FUS group as compared to TSL-Dox-only group (p < 0.05), providing supporting evidence on the therapeutic potential of the proposed strategy.

**Conclusions:** Our investigations demonstrated that spatially controlled thermal stress can be attained and sustained in the mouse brain, using a trans-skull closed-loop MRgFUS system, and used to promote the effective delivery of chemotherapy in gliomas from thermosensitive drugs. This system also allowed us to conduct mechanistic investigations that resulted in the refinement of our understanding on the role of thermal stress in augmenting mass and drug transport in brain tumors. Overall, our study established a new paradigm for effective drug delivery in brain tumors based on closed-loop ultrasound-mediated thermal stress and thermosensitive drugs.

## Introduction

Malignant glioma is the most common primary brain tumor with a 5-year overall survival below 10%, even when surgical, radiological, and chemotherapeutic interventions are applied 1. Despite the poor outcomes, there is a great interest in expanding the current chemotherapeutic interventions. This is because chemotherapy can penetrate the tumor better and tends to be less sensitive to tumor heterogeneity, which characterizes gliomas 2. It also provides unique opportunities for combinational approaches (e.g., immunotherapy, DNA damage repair inhibitors, etc.) 3,4. While, recent intensified chemotherapy protocols have shown promising findings 5, such approaches also lead to adverse systemic effects associated with nonspecific delivery, supporting the development of strategies to reduce systemic toxicity while attaining high drug accumulation to the tumor core.

Arguably, chemotherapy encapsulating nanoparticles can significantly reduce systemic toxicity by tailoring the nanoparticle properties (size, surface, etc.) to reduce uptake by sensitive organs (e.g., heart) and increase tumor accumulation either via the enhanced permeation and retention (EPR) effect or via active targeting 6. Unfortunately, their accumulation in brain tumors is not as effective as it is for some extracranial malignancies and in some cases it is lower compared to unencapsulated drug, resulting in sub-therapeutic drug doses 7-10. The reason for these dismal outcomes is manifold. While, the vasculature in gliomas is abnormally leaky and is frequently characterized by fenestrated endothelial cells and compromised tight junctions, its permeability is highly heterogeneous, and, as such, it is often considered a rate-limiting factor to effective drug delivery 11,12. In addition to heterogeneous vessel permeability, recent evidence suggests that solid stress, which is developed as the tumor grows in a confined environment, can compress the vessels and restrict blood flow and drug delivery in the tumor core 13.

Beyond the vascular barriers, nanoparticles larger than 60 nm will be confined to the abluminal side of the vessel wall as they will not be able to penetrate the brain interstitial space, which in mice, at least, is associated with pore sizes between 30 nm and 60 nm 14. The extravasated (i.e., abluminal side) nanoparticles will eventually release the smaller molecular weight drug into the tumor microenvironment via enzymatic degradation or other methods (long time scales: 4-12 h). However, the likelihood of the released drug to return to the circulation, which will now have very low drug concentration, will be at least equal to that of diffusing towards the tumor core. Hence, the net drug delivery and penetration to the tumor core, which is driven by drug concentration gradients, can be limited using standard nano-formulations.

Apart from the physical barriers to bulk transport, changes in the function of the dynamic influx/efflux transporter system at the vessel's luminal surface and cancer cell membrane may oppose both chemotherapy extravasation and cancer cell uptake 15. Although new and multifunctional nanoparticles are constantly being developed for improved drug delivery via active targeting 16, the requirement to concurrently surpass the aforementioned rate-limiting factors to clinically effective therapy, underscores the need for more robust drug delivery strategies against brain tumors.

Stimuli-responsive delivery systems provide a promising approach to locally improve chemotherapy delivery and penetration, while also reducing nonspecific toxicity 17,18. While there are several stimuli-responsive delivery systems, thermosensitive nano-formulations are of particular interest 19-22, as they can be combined with MR guided Focused Ultrasound (MRgFUS) technology. MRgFUS that is already used in the clinic for minimally invasive thermoablative interventions against neurological diseases 23 offers unique advantages for targeted drug release in brain tumors. Most notably it can target multiple small tissue volumes deep within the brain and through the skull via the use of multi-element phase arrays 24. The combination with MR Temperature Imaging (MRTI) allows monitoring and control of the temperature elevation in the focal region 25. A body of preclinical and clinical work in extracranial malignancies has shown that thermosensitive liposomes and localized mild hyperthermia (41 - 43 °C) with MRgFUS can enhance substantially the concentration and distribution of the chemotherapeutic agent (Dox) (see Table S1). This is primarily due to the higher concentration gradients between the blood and the tumor core (i.e., diffusive transport) that can be supported by the localized, rapid and control release mechanism. The observed improvement in the delivery of chemotherapy has led to increased median survival time (27 ± 12 days in FUS + drug group vs. 14 ± 6 days in drug only group) in a range of extracranial murine tumor models (see Table S1). It also showed improved Dox delivery in patients with hepatocellular carcinoma and encouraging responses in patients with local regionally recurrent breast cancer 26,27. Collectively, the demonstrated potential of combining these two technologies to attain high drug delivery while retaining low systemic toxicity, supports the assessment of this therapeutic strategy in brain tumors.

Beyond its ability to trigger chemotherapy release from thermosensitive liposomes, thermal stress creates unique opportunities to promote mass transport across the physical barriers and interfaces of solid tumors. Most notably, localized thermal stress at non-ablative thermal doses has been shown to reduce the interstitial fluid pressure (IFP) in extracranial tumors and improve nanoparticle accumulation 28,29. Thermal stress also appears to increase tumor perfusion and change vessel permeability 29,30. Moreover, hyperthermia can modify the properties of cancer cell membranes (e.g., cell membrane fluidity and heat shock protein production) 31, potentially enhancing drug uptake and creating several opportunities for synergies with other therapeutic interventions 32. While these observations highlight the different ways that non-ablative thermal stress can change the tumor microenvironment and augment mass transport in solid tumors, its role in modulating the cerebrovascular transport dynamics and drug delivery in the brain tumor microenvironment remains largely unexplored.

Taken together, the above investigations in extracranial malignancies suggest that combining the abilities of thermosensitive nanoformulations with those of MRgFUS-mediated hyperthermia can lead to a viable strategy for the targeted delivery of chemotherapeutics in malignant gliomas. Moreover, we posit that localized thermal stress can change the cerebrovascular transport dynamics in the brain tumor microenvironment to further improve drug delivery. To evaluate this therapeutic strategy and test our hypothesis, first, we developed a trans-skull closed-loop MRgFUS system for attaining controlled thermal stress in brain tumors in rodents. Then, we established safety thresholds and assessed the abilities of closed-loop trans-skull FUS-hyperthermia triggered release of Dox encapsulated by thermosensitive liposomes (TSL-Dox) for targeted and effective drug delivery in orthotopic glioma tumor models in mice and rats. Finally, we assessed the impact of thermal stress on the cerebrovascular transport dynamics in the glioma microenvironment using mathematical modeling and mass transport analysis based on dynamic contrast enhanced MRI (DCE-MRI) during the application of thermal stress in gliomas. As we elaborate below, our investigations not only demonstrate that closed-loop hyperthermia in combination with TSL-Dox can promote targeted and effective chemotherapy delivery in brain tumors but also allowed us to refine our understanding on the role of US thermal stress in modulating the vascular transport dynamics in the brain tumor microenvironment.

## Methods

### Transcranial hyperthermia FUS optimization

**Figure 1:**
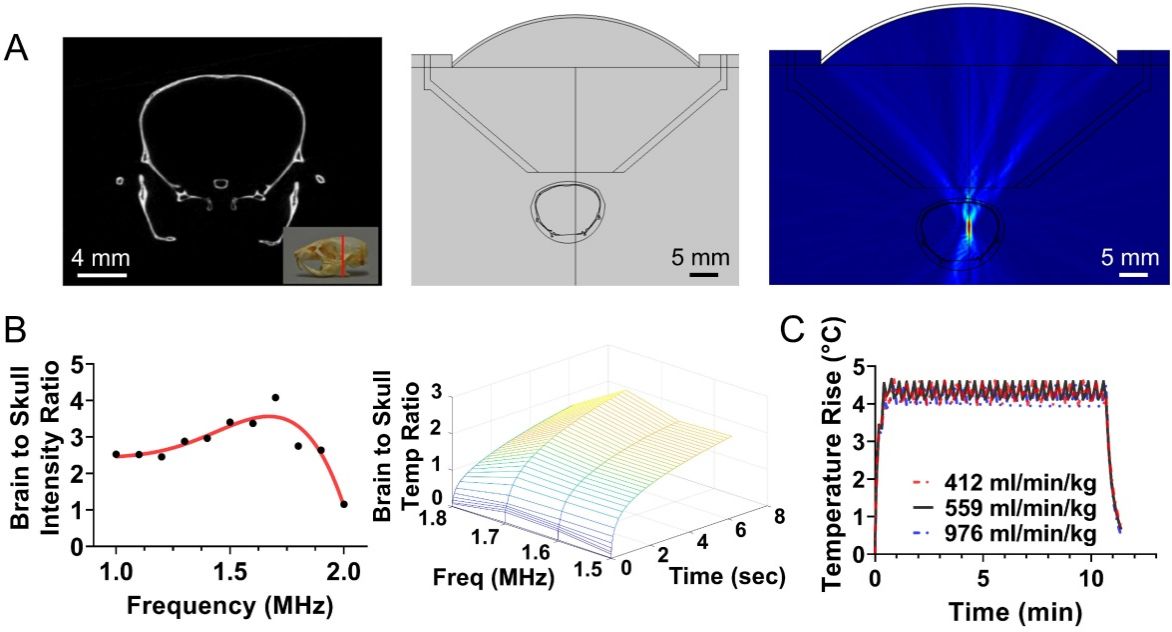
** Design of closed-loop MRgFUS system for localized transcranial hyperthermia in rodents. (A)** *Left*: CT scanned mouse skull with 50 µm voxel size; *Middle*: Simulation geometry based on the CT image of mice brain and the FUS system; *Right*: Simulated acoustic field (intensity) using FUS central of 1.7 MHz. **(B)** Brain to skull maximum acoustic intensity ratio plot and brain to skull maximum heat deposition extracted by the simulations for different frequencies. **(C)** *In silico* implementation of a simple binary (on-off) controller for different perfusion parameters incorporated to the Bioheat equation. The changes in the controller state were based on the MR thermometry frame rate, which currently is approximately 6 sec. These findings suggest that the proposed controller can maintain prescribed thermal stress for widely different tissue perfusion rates.

First, the feasibility of being able to perform trans-skull hyperthermia in small rodents was established. To do this, optimum frequency and geometrical characteristics of the FUS system using mathematical modeling was identified. A mouse skull (Skulls Unlimited, Oklahoma City, OK) was initially scanned with 50 µm voxel size using µCT (microCT50, Scanco) to create the model geometry. To simulate the experimental conditions, one of the CT scanned axial slice images (Burlington, MA, USA) along with the initial system design (Figure 1A) was imported into COMSOL and meshed using the physics-controlled routines of COMSOL. The model solved numerically the linear wave equation with heterogeneous speed of sound that was coupled to the bio-heat equation:


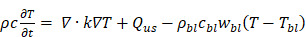
(1)

The different parameters in Eq. 1 along with the values used in the simulations are shown in Table 1. Based on this model the frequency of the FUS transducer was optimized by finding the maximum tissue to skull (instantaneous) acoustic intensity ratio as well as the maximum tissue to skull temperature ratio during a frequency sweep from 1 to 2 MHz.

### Thermosensitive Liposomal Dox (TSL-Dox) Preparation and Release Kinetics

Thermosensitive Liposomal Dox (TSL-Dox) was prepared as previously described 34. Briefly, lipids dipalmitoyl-sn-glycero-3-phosphocholine (DPPC), 1,2-distearoyl-sn-glycero-3-phosphoethano-lamine-N-PEG2000 (DSPE-PEG2000), and monostearoyl phosphatidylcholine (MSPC) (Avanti Lipids, Alabaster, AL) were dissolved in chloroform at a molar ratio of 85.7:9.7:5.0 (DPPC:MSPC:DSPE-PEG2000) and then dried under a stream of air to form a thin film of lipids. The vials were closed with a paper plug and placed in a desiccator overnight. The thin film was then hydrated with 300 mM citrate buffer (pH 4.0) at 55 °C until the lipids solubilized. Then the lipids were extruded five times through a 100 nm filter using a thermobarrell extruder (Lipex, Northern Lipids, Canada) at 60 °C. Dox hydrochloride (Sigma-Aldrich, USA) was dissolved in deionized water (2 mg/mL) and loaded into TSL at the final drug concentration of 2 mg/ml 34.

Temperature dependent drug release kinetics of these TSL was measured between 37 and 43 °C by a microfluidic device previously described 35,36. Briefly, TSL-Dox was diluted to 80 μg/ml in PBS at room temperature and was pumped through the microfluidic device consisting of a capillary tube, which was heated to the desired temperature. Concurrently fluorescent imaging was performed with a fluorescence imaging system (*in vivo* Xtreme, Bruker Biospin, Bellerica, MA, USA). While Dox fluorescence is quenched while encapsulated in TSL, fluorescence increases when Dox is released. As TSL-Dox pass through the heated capillary tube, the fluorescence signal increases during release allowing calculation of released fraction over time 34. For the experiments in rats (see below), the TSL-Dox formulation ThermoDox (Celsion Corp., NJ, USA) was used.

### *In vitro* cell drug uptake (nuclei) experimental procedures

To determine the rate of Dox binding to the nucleus the following *in vitro* experiments were performed. GL261 cells were seeded at a density of 20,000 cells/ml in cell imaging slides (Cell imaging coverglass, Eppendorf). At 60-70% confluency, free-Dox solution of 10 μM was applied to the imaging slide and fluorescence intensity was measured using confocal microscopy (LSM 710, Zeiss) every 5 min for 1 h. This concentration was selected because it produced images with high signal to noise ratio (SNR). In these measurements, the following 3 protocols were tested: 1) 37 °C, 2) Heat at 41.5 °C for 1 h, and 3) Heat at 41.5 °C (10min) + 37 °C (during imaging). The latter was a similar procedure as the one followed for the mouse studies.

### *In vitro* cell viability experimental procedures

The cell viability of GL261 glioma cells (TSL-Dox with and without hyperthermia) was assessed *in vitro* with the MTT assay (Cell proliferation kit, Abcam, USA). The cells were initially seeded in two different 96 well plates (Corning, USA) and cultured to reach 60-70% confluency in each well. The cells in both plates were then treated with treatment media prepared at varying concentration of the drug (from 0.01 to 100 µM) and incubated at 37 °C for an hour (total exposure time). Prior to the incubation, one plate was placed in a hot bath at 41.5 °C for 10 min to release the drug from TSL-Dox. Once the treatment was done, treatment media was discarded from both plates and the cells were incubated for another 23 h (24 h in total). MTT reagent and solvent were applied based on the assay protocol and the absorbance was read at OD = 590 nm using a plate reader. In all measurements the culture medium background absorbance was subtracted from the assay readings. Finally, using the corrected absorbance, the percentage cytotoxicity was calculated: % viability = 100 × ((control - sample) / control).

### *In vivo* experimental procedures

All animal procedures were performed according to the guidelines of the Public Health Policy on the Humane Care of Laboratory Animals and approved by the Institutional Animal Care and Use Committee of Georgia Institute of Technology. Genetically modified GL261 glioma cancer cells (100k) expressing firefly luciferase were stereotactically implanted into 6-8 weeks old female C57BL/6J mice (Jackson Laboratory) brain. The implantation site was located ~ 1 mm × 1 mm anterior and to the right of the bregma. A small hole was drilled at the implantation site and a syringe was inserted at 3mm depth to inject the cells. The cells were injected via slow infusion over 5 min. Tumor growth was monitored using T_2_ weighted Magnetic Resonance Imaging (MRI) using a Pharmascan 7T (Bruker) operating with under the Paravision 6.1 software environment. A standard TurboRARE sequence with fat suppression was used (TE = 35 ms, TR = 2.5 s, RARE factor = 8, the typical experiment consisted of 9 axial slices with a thickness of 1 mm). The tumor size was ensured to reach ~ 20-30 mm^3^ prior to each experiment. Using similar procedures, F98 glioma cancer cells (100k) were stereotactically implanted (2 mm × 2 mm anterior and to the right of the bregma) into 6-8 weeks old male Fischer rat (Charles River) brains.

**Figure 2:**
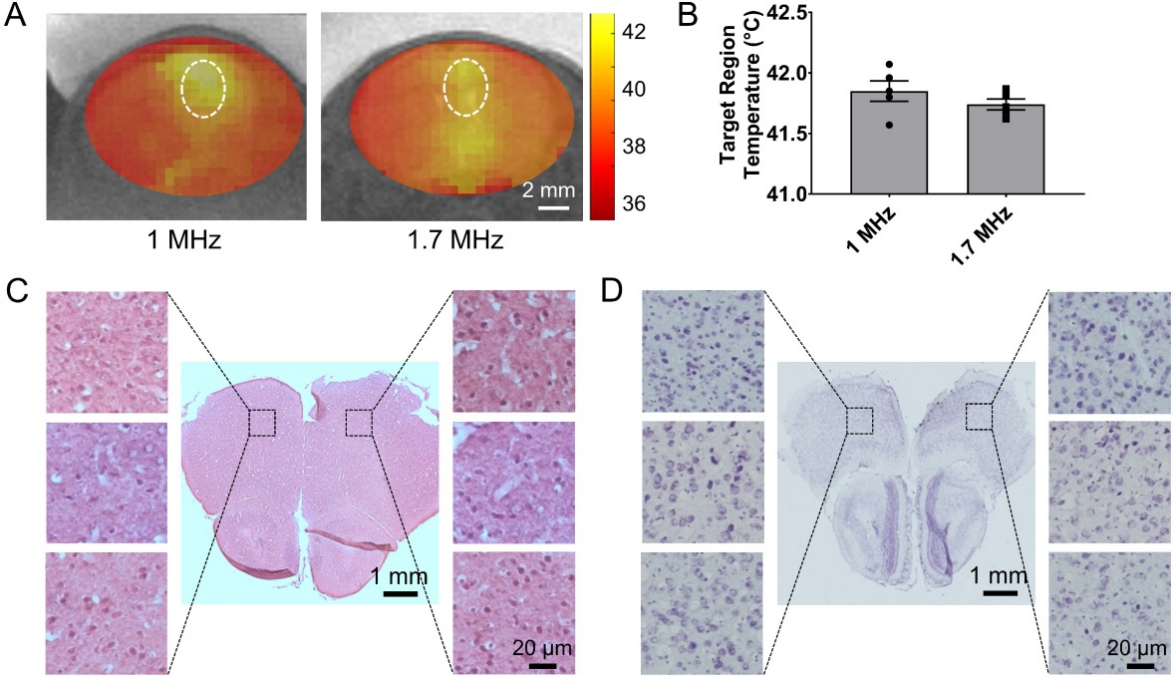
** Validation of closed-loop MRgFUS system for applying safely localized transcranial hyperthermia in rodents (A)** MR thermometry images (in Celsius) from experiments in healthy mice fused over T_2_ weighted images showing localized heat deposition using two different MRgFUS systems, with central frequency of 1 MHz and 1.7 MHz, respectively (5 mice per group). The FUS element had the same f-number. The white circle represents the targeted area. **(B)** Average focal temperature measurement (in Celsius) during transcranial hyperthermia experiment, showing narrower temperature ranges with the 1.7 MHz MRgFUS (SEM: 0.045 - 1.7 MHz vs. 0.084 - 1 MHz). **(C, D)** H&E and Nissl stained images of harvested brain tissues at 24 hrs (left - untreated, right - treated). Magnified images of 3 different samples are shown. The number of mice used per group was 3.

The sonications were performed with a custom-built MR guided FUS system (MRgFUS) consisting of an air-backed spherical shaped single element piezo transducer (see results section for additional information on the transducer and Figures 1 and 2) that was mounted to a manual 3D positioning system, which allowed sonications at different target locations. A water filled 3D printed cone was placed between the animal and the transducer for coupling. During the hyperthermia treatment, MR thermometry images were collected and used for monitoring the temperature at the targeted location using a transmit-receive surface MRI surface coil (Doty Scientific) that was attached to the 3D printed cone. Single FLASH images were measured repeatedly (TE = 5 ms, TR = 30 ms, flip angle = 30°, FOV, 40 × 40 mm^2^, slice thickness = 1 mm, the time to record and reconstruct one single frame was around 6.5 seconds). Once measured the images were accessed from a MATLAB (the Mathworks) program running on a remote computer, where images depicting a temperature changes were calculated using standard methods based on phase changes 37. The experiments in rats were performed using the same methods in a clinical GE 3T magnet.

A bistate (low/high states for below/above threshold temperature, respectively) closed-loop feedback controller that allowed to implement different mild hyperthermia protocols (10 min hyperthermia at 41.5 °C vs. 42.5 °C) was developed and evaluated using simulations and *in vivo* experiments. This feedback controller was controlled by temperatures extracted from a selected area of the MR temperature images. During hyperthermia 7 mg/kg of TSL-Dox for 5 min were administered intravenously and 45 min post administration the animals were sacrificed and the Dox delivery using fluorimetry and fluorescence microscopy was measured. For the control groups, the same dosage of TSL-Dox and free Dox (7 mg/kg) was administered.

To measure the K^trans^ value in tumor using Dynamic Contrast Enhanced MRI (DCE-MRI) the following parameter-selective images are needed: (i) a parameter selective image providing a map of T_1_ relaxivities, which is used to calculate the concentration of the contrast agent at a particular pixel, (ii) an image measured prior to the injection of a contrast agent to provide a baseline, and (iii) a series of images measured shortly after the injection of a bolus with contrast agent. (i) was achieved via a series of inversion recovery experiments (FLASH sequence preceded by an adiabatic inversion pulse (TE = 2.5 ms, TR = 1000 ms, flip angle = 30°, 3 slices with a thickness of 1 mm and a pixel resolution of 128 × 128). Six images with different inversion times [50, 100, 200, 300, 500, 700 ms] were recorded and a home-written code in MATLAB was used to calculate the T_1_-maps.

The K^trans^ images were measured under thermal stress. Prior to start of the DCE imaging acquisition, the focal temperature was maintained at the desired level (41.5 °C) for around 2 min using the closed-loop controller while collecting thermal MRI images. After that point DCE-MRI data were collected following the i.v. bolus administration of 8 μl gadolinium contrast agent (469 mg/ml, Magnevist). Images (iii) were collected to observe the entry of the contrast agent into regions of interest. This was achieved by a series of FLASH images (TE = 2.5 ms, TR = 20 ms, 3 slices, slice thickness = 1 mm, the time resolution was less ca. 7 sec/image). The first slice immediately recorded after the injection of the contrast agent served as baseline data (image type ii). During DCE-MRI acquisition the sonication power was set to a pre-determined level that was between the low- and high-levels of the controller for the rest of treatment.

The collected DCE-MRIs datasets were reconstructed and analyzed using a MATLAB code, which fits individual series of pixels using the public domain MATLAB function (fitdcemri) 38. The Calculation of K^trans^ images requires the arterial input function (AIF), describing the inflow of contrast agents. This data was calculated based on the selected ROI around the artery found in axial images and a Tofts model that was used to fit the DCE data 39. Our experimental setup consisting of a combined FUS system which operates in combination with a Tx/Rx surface coil had limited sensitivity impeding the determination of the AIF from arteries. Hence to improve the between-subject sensitivity in estimating the K^trans^ the AIF was individually measured using a conventional MRI coil (38mm coil, Bruker), where the arteries could be readily detected. The individually measured AIFs (n = 4) were then used to create the population average AIF, followed by fitting to a suitable AIF model. A bi-exponential model was applied to fit the data since this had been shown in previous investigations to provide the least variation in estimating the K^trans^ 40. While this approach might not be free of systematic errors it should allow to reliably detect differences in K^trans^ values in animals with and without thermal stress.

### Survival Analysis

**Figure 3:**
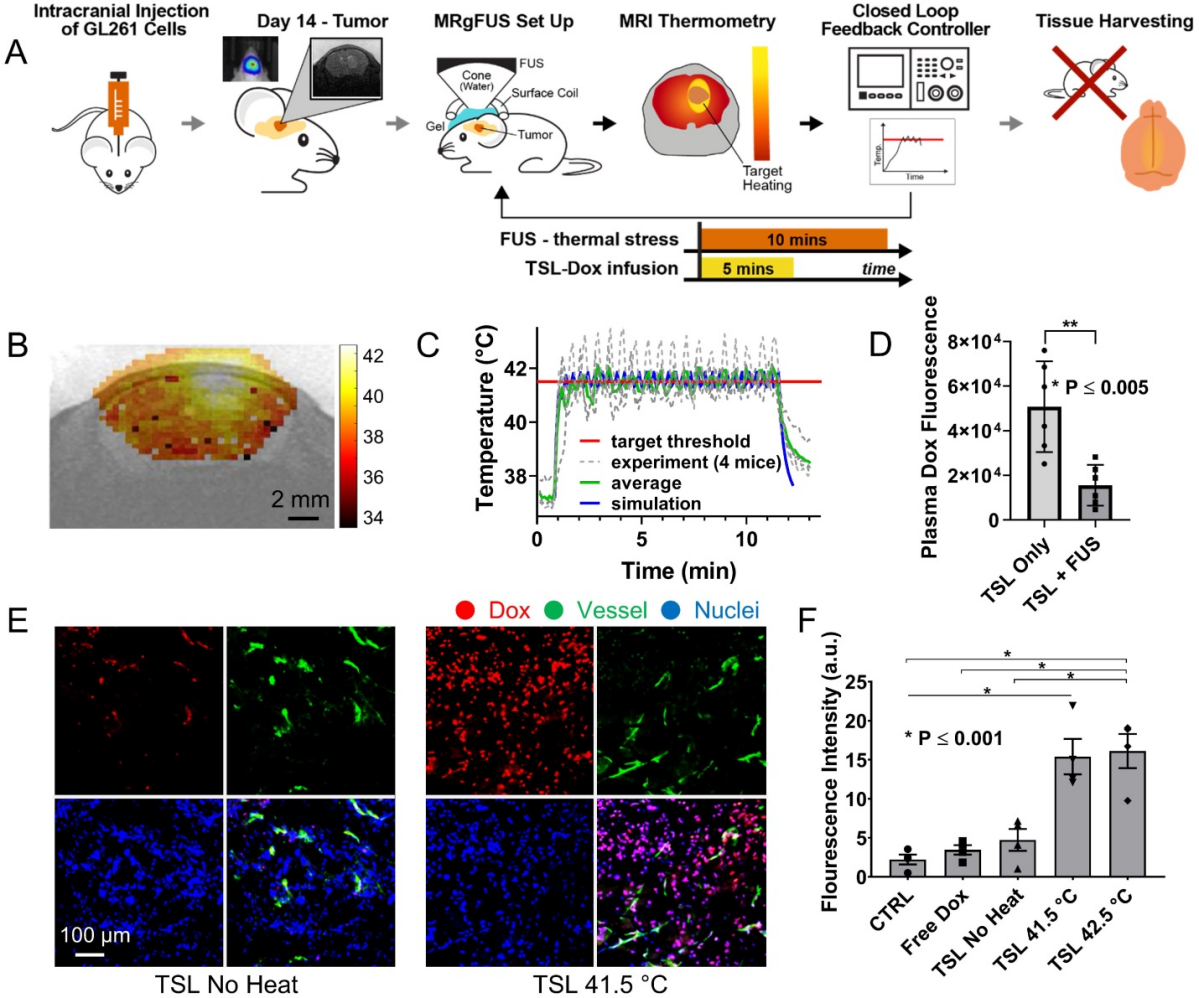
** Controlled Dox release from TSL-Dox with Closed-loop MRgFUS hyperthermia leads to improved Dox delivery in GL261 mice brain tumors. (A)** Schematic of the experimental protocol. **(B)** Thermometry image fused over MRI T_2_ image. **(C)** Temperature profiles of bistate closed-loop controller (low/high) from hyperthermia experiment demonstrating that all the MRgFUS treated mice reached the desired threshold for the duration of applied thermal stress for ~10 min. The experimentally determined controller temperature profiles were also in agreement with numerical simulations (41.5 ± 0.5 and 42.2 ± 0.4 °C). **(D)** Fluorometry comparison of remaining Dox in the blood after treatments, supporting that the employed protocol led to significant dox release (p ≤ 0.005, 6 mice per group). **(E)** Stained images of Dox, CD31, DAPI, and merged (No heat - left panel vs. Heat - right panel). **(F)** Quantification of the average fluorescence intensities between different treatment groups (p ≤ 0.001). The number of mice used per group was 4.

The therapeutic effect of the proposed treatment strategy was assessed in a survival study using the following two groups TSL-Dox with and without FUS-mediated hyperthermia. The treatment started when the tumor in each animal in the two groups reached 2 mm × 2 mm, as confirmed with T_2_-weighted MR images (i.e., the image slice with largest tumor segment). Each group, which consisted of 7 animals, was given 8 mg/kg of intravenous administration of TSL-Dox under anesthesia. FUS-mediated hyperthermia was applied for 10 min at 41.5 °C using the protocol shown in Figure 3A. Following the treatment, the tumors were imaged once a week with MRI. The animals were euthanized if they i) exhibited severely impaired activity, ii) weight loss exceeding 20% within one week compared to the baseline before the treatment, iii) tumor dimensions exceeding 4 mm × 4 mm (found in a T_2_-weighted image slice with largest tumor segment), or iv) treatment-related severe adverse events occurred that caused pain or distress and that could not be ameliorated with analgesics.

### Brain tissue processing

The animals that were euthanized at 1 h post drug administration were transcardially perfused with 20 ml of saline before harvesting the brains. The brains were fixed with 4% PFA overnight at 4 °C followed by 30% sucrose solution (4 °C) until it sunk to the bottom of the container. The brains were placed in O.C.T. compound and rapidly frozen to -80 °C. Subsequently, 30 µm sections were cut using a cryostat (*Leica 3050 S Cryostat*). For fluorimetric analysis of the tumor (rat), the brain was removed, and small tissue volumes (approximately 30 mg) identified by trypan blue staining were harvested along with their contralateral counterparts, which served as controls. The samples were soaked in acidified ethanol (50% ethanol in 0.3 N HCl). After homogenization with a tissue blender (Next Advance, Averill Park, NY) and refrigeration for 24 h at 4 °C, the samples were centrifuged at 16,000 g for 25 min. The fluorescent intensity of the supernatant was measured using a benchtop fluorometer (VersaFluor; Bio-Rad Laboratories, Hercules, CA; Ex/Em: 480/590 nm) 41.

### Immunofluorescence staining and microscopy

Tissues were first prepared for staining by fixing in 4% paraformaldehyde at room temperature for 10 min. After it was washed with PBS, the sections were first blocked for 1 h at room temperature (2% Bovine Serum Albumin, 5% goat serum in PBS). The sections were then incubated with primary antibody diluted in 1% Bovine Serum Albumin (1:100) for 12 h at 4 oC. Rabbit anti mouse CD31 (ab28364, Abcam Inc) was used for vessel staining. Next, the sections were incubated with donkey anti rabbit Alexa Fluor 555 secondary antibody diluted in 1% Bovine Serum Albumin (1:250, A31572, Invitrogen) for 1 h at room temperature. To stain the cell nucleus, samples were incubated with DAPI diluted in PBS (1:1000, 62248, Invitrogen) for 10 min after washing. Finally, the sections were rinsed with PBS to remove excess antibody, mounted with mounting medium (Prolong Glass Antifade Mountant, Lot# 2018752, Invitrogen), and covered with cover slips. The samples were cured with mounting medium for 24 h in the dark at room temperature before imaging. The sections were imaged with a 20× objective using a laser scanning confocal microscope system (LSM 700, Zeiss). The excitation wavelengths used for cell nucleus and vessel are 405 nm and 555 nm, respectively. The extravasation of Dox agent was measured using 488 nm excitation. To reduce interference from tissue autofluorescence we employed spectral imaging that allowed to remove the (background) tissue emissions from the Dox signal (See Figure S1).

### Fluorometry quantification of Dox in plasma

Plasma samples of animals from study groups were collected within 5 min post the treatment period and stored at -80 °C. A simple fluorometry quantification procedure of plasma Dox, as described elsewhere 42 was followed. A 30 μl of thawed plasma sample was added to 90 μl of PBS and 100 μl of 10% of Triton ×100 to release the liposomal Dox. The fluorescence of the plasma sample was measured via a fluorescence microplate reader (Synergy HT, Biotek Instruments Inc., Winooski, VT) with excitation and emission at 485 nm and 590 nm, respectively.

### Mathematical modeling of drug transport in brain tumor microenvironment

Immunofluorescence stained image was used to mimic and build a 2D tumor vascular network in our pharmacokinetic modeling 43. The mathematical model simulates the transvascular transport of drug across the endothelium and their transport in the interstitial space of a tumor along with the tumor cell uptake. Interstitial fluid was assumed to be homogeneous, Newtonian, and incompressible with constant viscosity µ. The transport of the interstitial fluid was modeled using Darcy's Law. The transport of drug in the interstitial space was simulated using a Convection-Diffusion-Reaction problem as follows:


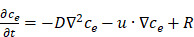
(2)

where 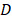 is the diffusion coefficient for corresponding domains (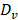 - vessel wall and 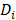 - interstitium space), 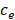 is the drug extracellular concentration, and R is the reaction term, which is given by the following equation in the interstitial sub-domain:


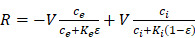
(3)

The drug in the cellular compartment (*c_i_*) as well the drug bound to the nucleus (*c_b_*) are modeled by the following ordinary differential equations:


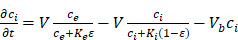
(4)


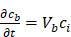
(5)

The intersitium permeability is given as the brain kinematic viscosity multiplied by the interstitium hydraulic conductivity (*K*). The parameters in *Eqs*. (2) - (5) are summarized in Table 2 and a summary of the boundary conditions used in each subdomain in the model are detailed in our previous study 43.

In our simulations, the rate of transvascular fluid transport is modeled using Starling's law that ignores osmotic pressure difference 44. The rate of transvascular fluid flow is defined as:


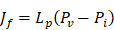
(6)

where 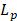 is the hydraulic conductivity of the vessel wall, 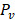 and 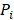 are vascular and interstitial pressure respectively. To simulate the mass transport across the vessel wall, the Péclet number across the vessel wall, 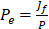 with 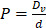, where *d* is the vessel wall thickness, was calculated. The rate of drug transvascular transport 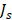 across the vessel wall is modeled using Starling's approximation 44. When the Péclet number is less than or equal to 1, the Kedem-Katchalsky equation is used:


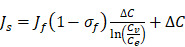
(7)

where 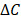 is the anticancer agent concentration difference across the vessel wall, and 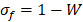 with W being the convective hindrance factor. When the Péclet number is greater than 1, the Patlak equation is used:


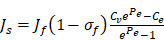
(8)

For the boundary condition, the agent-specific concentration profiles were applied at the vessel wall domain with TSL-Dox released drug concentration.

In order to incorporate thermosensitive liposomal Dox release mechanism into the model, the following ODE equation that provides the rate of change of thermosensitive liposomal Dox (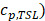 within the systemic plasma is used 45, in addition to the governing equations described above:


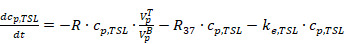
(9)

where the first and the second terms account for the decrease of liposomal drug caused by the drug release during heating in the tumor plasma and the release of drug which takes place at 37 °C body temperature in the systemic plasma. 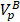 is the total plasma volume in the body and 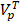 is the plasma volume in the tumor region. The last term accounts for the body clearance of liposomes with 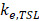 being the transfer constant for clearance of TSL from the systemic plasma. Based on the different thermal stress condition with temperature (heat vs. no heat), different plasma drug concentration profiles were generated (Figure S2). The profiles were modified accordingly to the model with different treatment conditions (thermal exposure fractionation). The rate of release of Dox from the liposomes depends on the local temperature (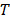). The release rate (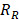) of Dox from the TSL was determined as follows: a bi-exponential fit of the experimental data of the time course of Dox release fraction (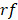) at temperatures between 37 and 47 °C. The amount released depends on the residence time (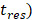 of the TSL in the heated region; 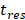 in our model is equal to 1/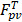, i.e., its change depends on local perfusion (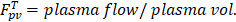). The release rate 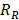 is then:


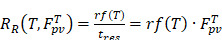
(10)

It is noted that there will still be some amount of Dox released in the systemic plasma throughout the simulation since the release fraction *rf* (*T*) at body temperature (37 °C) is non-zero. Using the amount of Dox released by the thermosensitive liposomal Dox in systemic plasma, the overall rate of change of free Dox concentration (*c_p_*_,Dox_) in the systemic plasma is then estimated as follows:


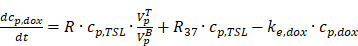
(11)

where the last term accounts for the body clearance of free Dox with *k_e,dox_* being the transfer constant for clearance of free Dox from the systemic plasma. The agent-specific concentration profiles applied at the vessel wall domain with TSL-Dox released drug concentration in systemic plasma are shown in Figure S2.

The parameters in Table 2 were found as part of a model identification procedure that solves an optimization problem in which the model was combined with the Nelder-Mead method and updated with respect to the system parameters until the experimental and numerical curves (i.e., objective function) were matched (Figure S3). The constraints to this optimization problem were provided by experimental measurements of the cellular uptake rate (*in vitro*) and the Dox fluorescence intensity measured at a specific distance (15 um) from the vessel wall (*in vivo*) (Figures S4 and 5A). The measured data of uptake rate was fitted to specific one site binding equation to generate a smooth objective function that reaches desired Cb concentration. The initial values for the model parameters were taken from the literature for Dox. The obtained concentration with transient cellular uptake kinetic is provided as a least-squares objective, which is then compared to the output of the model at the same measured distance. The initial values for the model parameters were taken from the literature (see Table S2).

Using the refined parameters of the mathematical model, a local sensitivity analysis to identify the rate-limiting factors of the system and evaluate different treatment protocols was performed. In the sensitivity analysis the amplitude of each system parameter was varied by ±25% and determined the drug bound to the nucleus (*c_b_*). Then, the derivative of the nucleus binding concentration *c_b_* with respect to any parameter P_i_, 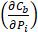 was approximated and a normalization to the sensitivity 
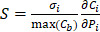
 was introduced to compare the sensitivities to different parameters. In this relationship 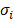 is the standard deviation of *P_i_* across the repetitions of each experiment and max(C_b_) is the peak nucleus bound concentration measured.

### Statistical analysis

Results are expressed as mean ± SD or SE. The statistical significance of data was assessed using Student's t test and ANOVA test; p < 0.05 was considered statistically significant in all analyses. The tumor volumes at the beginning of the survival study were compared using one-way ANOVA test. The Kaplan-Meier method was used to compare survival of animals in each group. Significance was calculated by using log-rank test with Yates' correction. All statistical analyses were performed using GraphPad Prism.

## Results

### Development of closed-loop MRgFUS system for localized transcranial hyperthermia in rodents

To apply localized US thermal stress (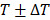 with Δ*T* < 0.5 °C for prolonged times *t* > 1 *min*) to brain tumors in rodents we developed a closed-loop trans-skull MR guided focused ultrasound system. For optimal operation, this system needs to attain a desired temperature inside the brain while minimizing skull heating. To identify the optimal FUS frequency and geometrical characteristics for trans-skull hyperthermia in rodents we employed mathematical modeling of ultrasound propagation in heterogenous viscous media (i.e., skull; Figure 1A) and coupled it with the bioheat equation. The acoustic properties for the skull (density and speed of sound) were extracted by semi-empirical models based on CT data of the mouse skull 46. After we set up the model using a focused transducer (F-number 0.75) with its focus placed inside the brain (right hemisphere; Figure 1A), we identified the frequency (≈ 1.7 MHz) that resulted in maximum brain to skull (instantaneous) acoustic intensity ratio and maximum brain to skull temperature ratio (Figure 1B). Using this frequency, we implemented *in silico* a binary (on-off) controller and confirmed that the proposed system could sustain specific thermal stress for a wide range of physiologically relevant tissue perfusion properties (Figure 1C).

Based on these findings we built and evaluated experimentally in healthy rodents an MR-guided FUS-system (central frequency 1.7 MHz) for closed-loop localized trans-skull hyperthermia. Our experimental findings, using a bistate controller (low/high states for below/above threshold temperature, respectively), indicate that our design had the desired performance (ΔT < 0.5 °C). To confirm the optimal response of the proposed system design we compared our findings with an MRgFUS system with a central frequency of 1 MHz. As shown in in Figure 2A, the MRgFUS system with 1.7 MHz central frequency resulted in better penetration through the skull, focal temperature closer to the intended temperature (41.5 °C), and lower variation in the focal temperature (SEM 0.045 vs. 0.084) as compared to the 1 MHz system (Figure 2B). These data confirm our numerical investigations and demonstrate that an optimal frequency does exist for applying localized thermal stress in mice brain without overheating the skull. Twenty-four hours after treating with FUS hyperthermia, we harvested the brain and assessed the tissue with H&E and Nissl staining (Figure 2C-D). Both the H&E and Nissl-stained images showed no signs of damage associated with the applied US thermal stress as compared to contralateral brain side (non FUS targeted). These data provide evidence that the proposed closed-loop MRgFUS system can safely apply localized thermal stress (41.5 ± 0.5 °C for 10 min) in the brain of mice (see also Figure S5).

### Controlled Dox release from TSL-Dox with closed-loop MRgFUS hyperthermia leads to improved Dox delivery in orthotopic glioma tumors in rodents

After establishing the optimum MRgFUS design and verifying that trans-skull MRgFUS can be used to safely apply mild hyperthermia in the brain, we assessed the potential of localized thermal stress to trigger the release of Dox from thermosensitive liposomes (TSL-Dox) and facilitate localized delivery in brain tumors. For our investigations, we employed the experimental protocol shown in Figure 3A. After we confirmed that mild hyperthermia (41.5 ± 0.5 °C for 10 min) can be accurately applied to the GL-261 mice glioma tumors (Figure 3B-C) we quantified the Dox release and cellular uptake in the tumor microenvironment. The controls and treated groups used in this study were the following: i) No Dox - Control, ii) Free Dox, iii) TSL-Dox - No FUS, iv) TSL-Dox + FUS at 41.5 °C for 10 min, and v) TSL-Dox + FUS at 42.5 °C for 10 min. Forty-five minutes after the treatment, we sacrificed the animals and analyzed the Dox release and delivery. Fluorometric comparison of the Dox remaining in the blood after the treatments (Figure 3D) demonstrates significantly lower Dox in the circulation after the application of mild hyperthermia (p ≤ 0.005, core body temperature remained at 37.7 °C), providing evidence that the employed protocol leads to significant Dox release from TSL-Dox within the vasculature. Analysis of the tumors indicted that the TSL-Dox + FUS groups (41.5 °C and 42.5 °C) had significantly higher Dox penetration and cellular uptake as compared to control and non-FUS groups (Figure 3E-F) (~ 3.5-fold increase, p < 0.001; both for 41.5 °C and 42.5 °C). Although TSL-Dox + FUS protocols showed similar Dox delivery, the harvested tissues post-FUS indicated that mild hyperthermia at 42.5 °C for 10 min induces a significant hemorrhage in the brain near the skull surface (Figure S6). Hence, to safely apply mild hyperthermia in brain tumors in mice through the skull mild hyperthermia should be limited to 41.5 °C (for 10 min). Closer inspection of the fluorescence microscopy images indicates that in the “TSL-Dox only” group Dox is primarily localized in the vessel wall (CD31 positive) and is bound to endothelial cells, demonstrating that without the application of hyperthermia TSL-Dox cannot penetrate the tumor vasculature (Figure 3E - left). On the other hand, in the TSL-Dox + FUS (41.5 °C for 10 min) group a significant improvement in Dox penetration can be observed.

**Figure 4:**
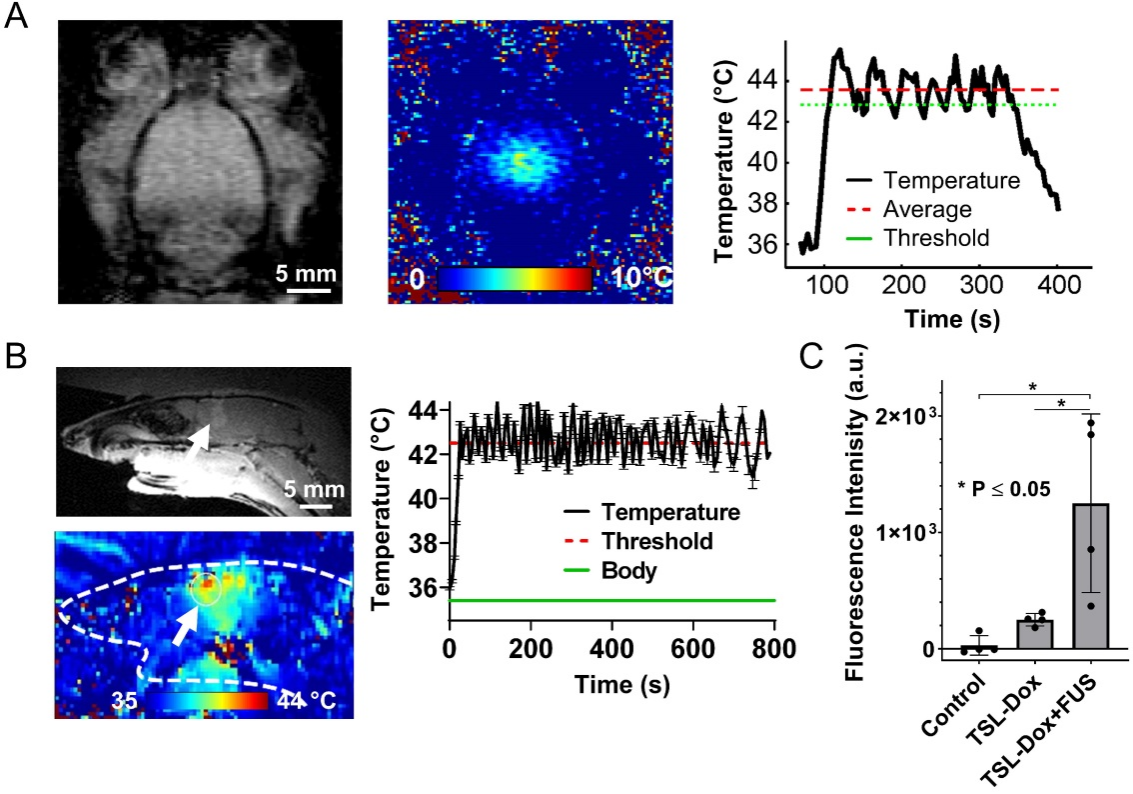
** Controlled Dox release from TSL-Dox with closed-loop MRgFUS hyperthermia in a F98 rat brain tumor. (A)** Data from a representative experiment during FUS-induced mild hyperthermia in healthy rats using MR thermometry-based closed-loop controller (42.5 ± 1 °C). The controller was able to reach the threshold temperature (42.5 °C) within few seconds and remain at it for several minutes (±1 °C). It was found that cooling the water below 15 °C can eliminate brain damage due to skull-heating. **(B)** *Top*: representative contrast enhanced MRI (T_1_ weighted) image of the F98 tumor in rat. *Bottom*: representative MR thermometry image during the application of closed-loop FUS-induced mild hyperthermia the F98 tumor in rat. **(C)** Comparison of average fluorescence intensities between different treatment groups: i) Control - No Drug, ii) TSL-Dox - No Heat, iii) TSL-Dox + Heat at 42.5 °C) (p ≤ 0.05). The number of rats used per group was 4.

To assess the robustness of the proposed drug delivery strategy and confirm its application to a larger animal model we tested the proposed protocol in orthotopic F98 glioma rat model. As shown in Figure 4A (healthy rats), we were able to reach the threshold temperature (42.5 °C in this case) within a few seconds and remain at it for several minutes (± 1 °C). Because the body temperature in rats was lower than that of mice (35 °C versus 37.7 °C) and the rat brain is significantly larger, there was a smaller overlap of the FUS focal region with the skull, enabling us to employ a higher threshold temperature (≈ 42.5 °C). Despite the higher temperature threshold employed, we did not observe any tissue damage (after gross tissue inspection). Moreover, fluorometric assessment of excised F98 glioma tumors demonstrated significantly higher doxorubicin delivery under FUS-hyperthermia (Figure 4C; over 5-fold increase as compared to control, TSL-Dox only; p < 0.05), supporting the robustness of our findings and of the proposed targeted drug delivery strategy in gliomas.

### Integration of experimental measurements with physiologically based pharmacokinetic modeling reveals that localized thermal stress increases the brain vessel diffusion coefficient in the GL261 mice brain tumors

**Figure 5:**
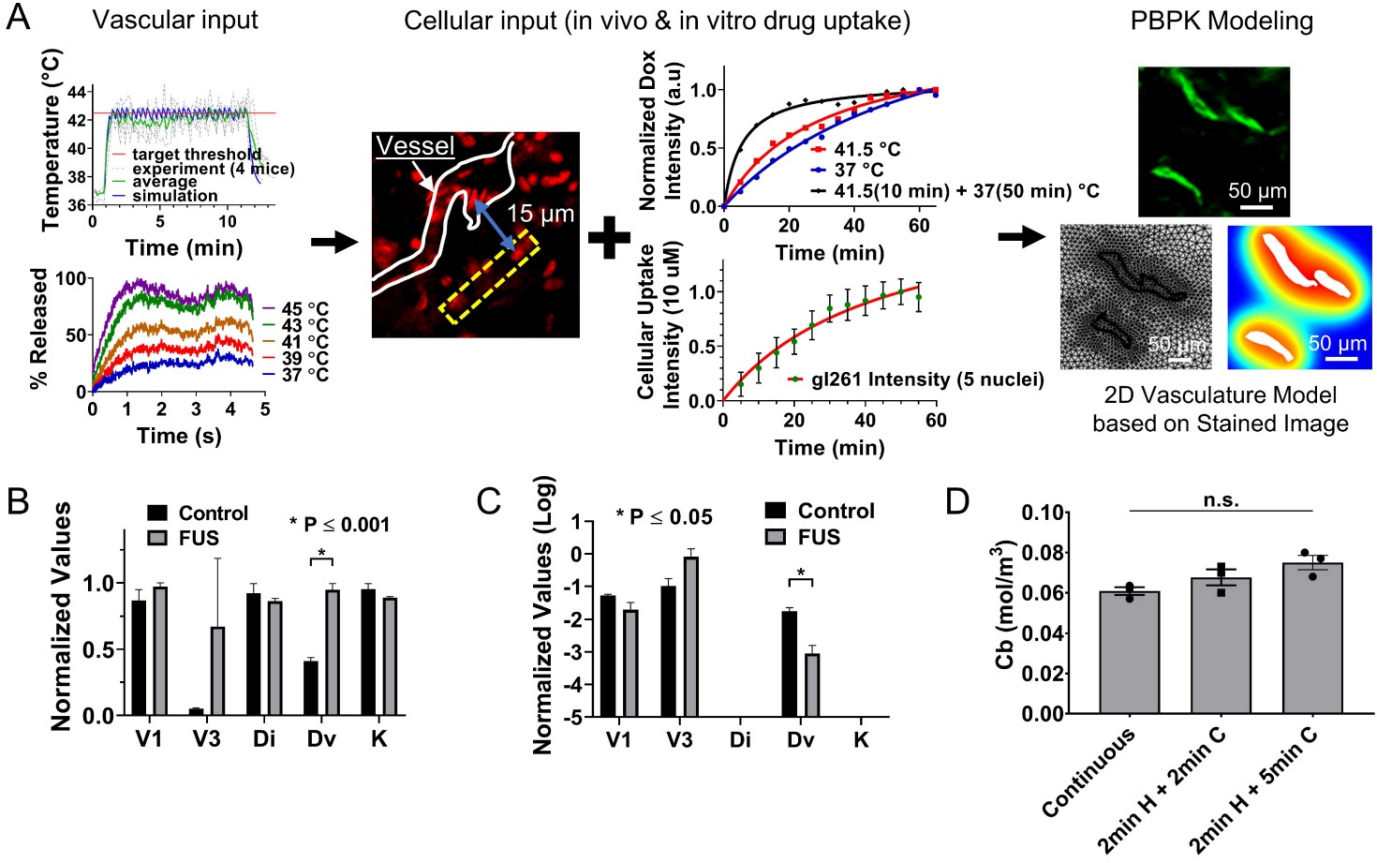
** Physiologically based pharmacokinetic modeling suggests that thermal stress increases transvascular transport in the GL261 mice brain and that thermal exposure fractionation can improve the amount of drug delivered to cancer cells. (A)** PBPK model building based on *in vitro* and *in vivo* measurements. *In vitro* measurements included the temperature thresholds for Dox release and the rate of Dox uptake by the GL261 cell lines. *In vivo* measurements included the focal temperature and percent drug release profile (vascular input) the drug uptake and penetration in the GL261 tumor model (cellular input) and vessel geometry (PBPK modeling geometry) based on the immunofluorescence staining. The computational domain was obtained by the segmented vessel that was meshed using physics-controlled routines in COMSOL Multiphysics (right panel). On this geometry, we applied a pharmacokinetic model that could capture the transport of drug across the vessel wall, in the interstitial space and into the cells and nuclei. Subsequently, we identified the model parameters by minimizing the differences between the model output and experiment specific measurements determined by the *in vitro* (rate of uptake) and *in vivo* (relative level of uptake) quantification of cellular drug uptake (i.e., objective function). A good agreement between model output and the recovered time dependent objective functions were observed (Figure S3), indicating that the underlying assumptions were reasonable. **(B)** The inferred model parameters for non-FUS and FUS cases were extracted using the proposed system identification procedures (Table 3). Only the vessel effective diffusion coefficient was found to be significantly different across the two different groups (p ≤ 0.001). **(C)** Sensitivity analysis based on the refined PBPK model showing that the thermal stress reduces the relative importance of the transvascular transport in gliomas. The values for Di and K are substantially lower compared to other parameters. **(D)** Applying thermal stress at different regimens (i) 10 min heating, ii) 2 min heating followed by 2 min cooling and repeated 5 times, iii) 2 min heating followed by 5 min cooling and repeated 5 times) improves the amount of the delivered drug in the cancer cell nucleus (Cb).

Although the experimental data demonstrated that localized thermal stress combined with TSL-Dox is a viable strategy for systemic drug delivery in gliomas, we postulated that physiologically based pharmacokinetic (PBPK) modeling could be used to better understand the transport dynamics in the tumor microenvironment (e.g., BBB permeability, interstitial transport, cancer cell uptake). Hence, we first set up a PBPK model based on the experimentally determined geometry (Figure 5A), next we inferred the PBPK model parameters (Table 3) using system identification methods based on experimental measurements (*in vitro* and *in vivo*) of Dox release and delivery with and without applying thermal stress (Figure 5A-B), and finally we conducted parameter sensitivity analysis to identify the rate-limiting factors of the system (Figure 5C). Interestingly, the identified model parameters for the FUS and the non-FUS groups (Figure 5B and Table 3) indicated significant differences only for the vessel effective diffusion coefficient, Dv, (2.3-fold increase, P ≤ 0.001). These data apart from providing a quantitative assessment of the transport properties of brain tumors are the first to suggest that thermal stress can significantly increase the vessel permeability in the brain tumor microenvironment.

As we alluded to, to obtain further insights into the transport dynamics of the system, identify system (transport) parameters with the highest impact on mass transport and drug delivery, and establish optimal treatment protocols, we performed a sensitivity analysis based on the refined PBPK model. Our results indicate that the thermal stress reduces the relative importance of the transvascular transport (i.e., vessel effective diffusion coefficient, D_v_) for effective drug delivery in gliomas (Figure 5C). They also suggest that overcoming trans- and inter-cellular transport dynamics are critical for attaining effective drug delivery in gliomas. Next, we tested if different hyperthermia treatment regimens, such as thermal exposure fractionation, for the same total heat duration, would impact the amount of drug delivered to cancer cells. Interestingly, mathematical inference suggests that applying thermal stress for 2 min followed by 5 min cooling in 5 cycles does affect the amount of delivered drug if the TSL-Dox plasma half-life is longer than the total duration of the treatment (Figure 5D). This has important clinical implications, as this treatment protocol (i.e., with intermittent cooling) could be more readily implemented in humans, where skull heating could limit the thermal stress that can be applied continuously to the tumor.

### Closed-loop MRgFUS transcranial hyperthermia leads to significant improvement in the K^trans^ and higher free Dox delivery in the GL261 mice brain tumors

**Figure 6:**
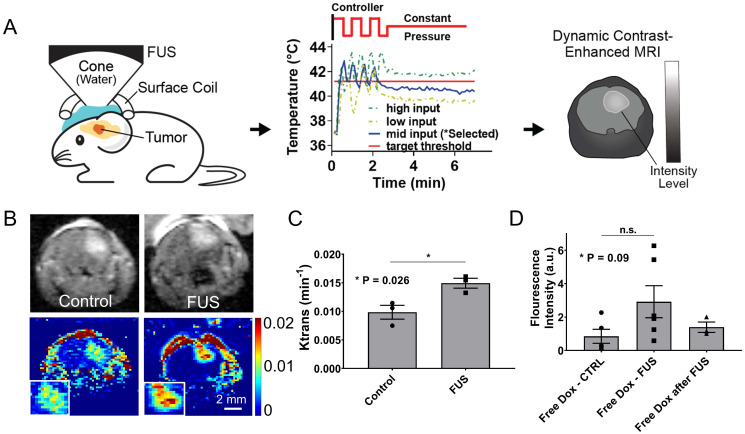
** Closed-loop MRgFUS transcranial hyperthermia leads to increased K^trans^ and free Dox delivery. (A)** Schematics of K^trans^ experimental protocol with FUS input pressure determination for optimized temperature threshold inside the brain. **(B)** *Top:* Representative FLASH MRI images of tumors with and without FUS recorded shortly as part of an image series after the bolus administration of 8 μl gadolinium contrast agent (469 mg/ml, Magnevist). *Bottom*: Calculated K^trans^ map of tumors with and without FUS. **(C)** Quantification of K^trans^ between the control and the FUS group, showing significantly higher K^trans^ value in the FUS treated tumor (p = 0.026). The number of mice used per group was 3. **(D)** Fluorescence intensities of free Dox in tumor regions were compared between different treatment groups: i) Free Dox (control), ii) Free Dox was injected during the application of thermal stress, and iii) Free Dox was injected immediately after the DCE-MRI databases were collected (post-FUS). The application of hyperthermia resulted in an upward trend in the Dox accumulation in the tumor, albeit this trend was not statistically significant (p=0.09). The number of mice used per group was ≥ 4.

To prospectively validate our experimental observations and numerical predictions related to the changes in the transvascular transport we measured the changes in K^trans^, which provides a measure of vascular permeability, using dynamic contrast enhanced MR imaging (DCE-MRI) during FUS-hyperthermia in the GL261 glioma mouse tumors. Based on the experimental protocol shown in Figure 6A, after two minutes of mild hyperthermia using closed-loop MRgFUS, we applied a constant pressure level (between the high and low states of the controller) that based on prior experiments was able to keep the temperature level close to 41.5 °C (Figure 6A). This modification to the experimental protocol was essential in order to measure the K^trans^ value (DCE-MRI datasets) during the application of thermal stress, where we hypothesized that it would have its highest value. As evidenced by our experimental findings (Figure 6B-C), the measured K^trans^ values were significantly higher in the FUS group as compared to the no FUS group (0.0097 vs. 0.0148 min^-1^, p = 0.026), demonstrating that thermal stress can change the transvascular transport dynamics in brain tumors. This observation, which supports our previous findings (Figure 5B), suggests that the observed increase in drug uptake in the TSL-Dox + FUS group can be the result of the combined effects of localized Dox release and thermal stress-mediated changes in the transvascular transport in the glioma tumor microenvironment.

To further understand the impact on drug delivery of the thermal stress-mediated increased transvascular transport in glioma tumors, we applied controlled hyperthermia, as before (i.e., Figure 3B), but instead of using TSL-Dox we used free (un-encapsulated) Dox. Interestingly, US thermal stress combined with free Dox resulted in ~ 1.6-fold (p = 0.09) increase in Dox delivery in the GL261 tumors as compared to the non-FUS group (free Dox) or the group that Dox was delivered shortly after the application of thermal stress (free Dox after FUS; Figure 6D). While this improvement is substantially lower to the delivery attained with the TSL-Dox + FUS, our findings demonstrate that thermal stress can promote acute changes in the transvascular transport dynamics in glioma tumors and lead to improved drug delivery.

### Controlled Dox release from TSL-Dox with closed-loop MRgFUS hyperthermia leads to improved survival in the GL261 tumor-bearing mice

**Figure 7:**
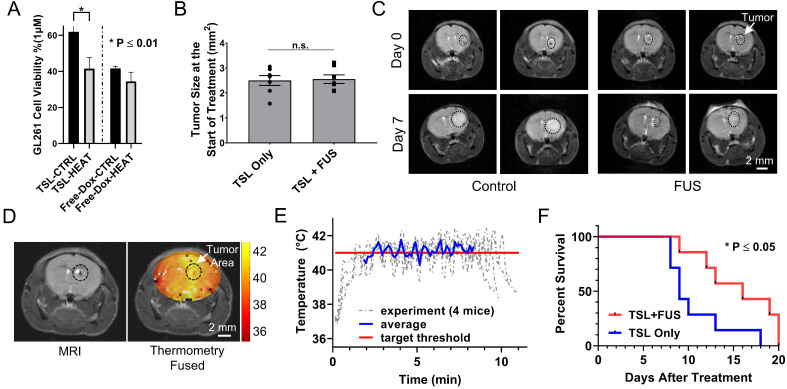
** Therapeutic efficacy of TSL-Dox with and without closed-loop FUS hyperthermia. (A)** Analysis of the GL261 cell viability (*in vitro*) between TSL-Dox with and without hyperthermia (10 min at 41.5 °C).** (B)** Estimation of tumor growth based on T_2_ weighted MRI images taken on the treatment day.** (C)** Representative MRI images of control and FUS groups (2 mice from each group) with outlines (black circle) showing the tumor growth (treatment day - top vs. post 7 days - bottom). **(D)** Thermometry image fused over a T_2_ weighted MR image. **(E)** Temperature profiles of bi-state closed-loop controller (low/high) from hyperthermia experiments demonstrating that the MRgFUS treated mice reached the desired threshold for the duration of applied thermal stress (~10 min). **(F)** Survival analysis in GL261 tumor mice (TSL-Dox with and without hyperthermia at 41.5 °C for 10 min) (P = 0.032). The increase in overall survival in the TSL-Dox with hyperthermia was 15.6 ± 4.0 days (median = 16) and in the TSL-Dox alone was 10.7 ± 3.4 days (median = 9) from the day the treatment started. The number of mice used per group was 7.

To assess the therapeutic efficacy of the proposed strategy we conducted a survival study using the following two groups TSL-Dox with and without FUS-mediated hyperthermia. We first assessed *in vitro* the differences in the GL261 cell viability between the two groups. While the IC_50_ indicated that this cell line is not very sensitive to Dox (Figure 7A; IC_50_ ≈ 1 µM), it did result in a significantly lower cell viability in the presence of hyperthermia (P < 0.01). The latter provided sufficient justification for testing the therapeutic potential of this strategy with this cell line *in vivo*. In the survival study, we employed the same Dox dose (8 mg/kg) used in previous investigations that assessed the therapeutic efficacy of microbubble enhanced FUS in combination with free Dox using the same tumor model 47. In our experiments we also accounted for tumor size among the two different groups and made sure that it was similar to previous investigations (Figure 7B and C) 47. During the sonications, the applied thermal stress within the tumor area (41.1 ± 0.52 °C) and among the different animals (41.3 ± 0.43 °C) was consistent and within the targeted level (Figure 7D and E). The survival analysis indicates a significant improvement in the survival in the group with TSL-Dox + FUS-hyperthermia as compared to TSL-Dox only (Figure 7C and F; p = 0.032). The log rank test revealed a 78% greater median survival after TSL-Dox with FUS-hyperthermia in comparison to TSL-Dox alone. The improvement in survival is also consistent with the GL261 cell viability between TSL-Dox with and without hyperthermia (10 min at 41.5 °C) we observed *in vitro*. Overall, the survival study confirmed the therapeutic potential of the proposed treatment strategy against brain tumors.

## Discussion

Stimuli-responsive delivery systems such as thermosensitive liposomes represent a promising strategy to locally enhance drug delivery while maintaining low systemic toxicity. In this study, we assessed the combined abilities of closed-loop trans-skull MRgFUS-hyperthermia with those of chemotherapy encapsulating thermosensitive nano-formulations to improve the systemic delivery of chemotherapy in malignant gliomas. Collectively, our findings demonstrated that FUS-mediated hyperthermia combined with TSL-Dox can improve substantially Dox accumulation and uptake in two glioma tumor models in rodents (mice and rats) and lead to statistically significant improvement in survival (mice). Our results also suggest that FUS-mediated thermal stress can trigger acute changes in the cerebrovascular transport dynamics in the brain tumor microenvironment and lead to improved transvascular transport without damaging the brain. Together, our mechanistic investigations allowed us to establish a new paradigm for effective and targeted drug delivery in brain tumors based on closed-loop ultrasound-mediated thermal stress and thermosensitive drugs.

To implement the proposed therapeutic strategy, we designed a closed-loop MRgFUS system that was able to generate controlled and localized thermal stress in the brain (Figures 1 and 2). Attaining controlled thermal stress through the skull is not a trivial problem, especially in mice. For example, although at low frequencies (< 1 MHz) most of the energy is transmitted through the skull, the resulting large focal region overlaps substantially with the skull, which due to its higher absorption leads to disproportionally high skull heating (See Figure S7). On the other hand, at higher frequencies (> 2 MHz) skull reflections and aberrations become significant, and thus limit our ability to focus the beam in the brain through the skull (See Figure S7). Using a physically accurate numerical modeling, we were able to identify and validate experimentally the optimal frequency for this system (Figure 2A). Although we did not conduct extended experiments using the 1 MHz transducer, as it is evident from Figures 2A and 7D, operation at optimal frequency (≈ 1.7 MHz) ensures both reproducible experimentation and heating the entire tumor at the desired temperature. The ability to diminish the temperature difference between the skull and the tumor (assuming the skull temperature is higher than the focal temperature) also allows maintaining the thermal dose throughout the entire brain at safe levels (Figure 2). Note that if the skull temperature is only 2 °C above the tumor (43.5 °C versus 41.5 °C) the thermal dose for 10 min will be more than order of magnitude higher (14 min ECM versus 1.25 min ECM), which in turn leads to thermal doses very close to accepted limits for brain tissue damage (CEM_43_ ≈ 20 min) 48.

In addition to identifying the optimum frequency for the FUS system, we also developed a bistate controller that allowed to consistently maintain the thermal dose well below the limits for brain tissue damage (CEM_43_ < 20 min 48; Figure 2). In addition to monitoring and controlling the focal heating, the total treatment needs to be relatively short (10 min at 41.5 °C in mice) to remain within the safety limits. Interestingly, mathematical inference suggests that thermal dose fractionation using ultrasound burst cycles, as opposed to continued wave US exposure, for the same total heat duration, leads to improved drug delivery. The latter is in agreement with recent experimental studies in extracranial tumors that demonstrated that ultrasound burst cycles designed to release the drug at multiple brief periods can lead to significant improvement in overall Dox delivery 49,50. While more investigations are needed to establish optimal volumetric sonication schemes for limiting off-target brain tissue damage and attaining improved Dox delivery in brain tumors, our findings indicate that closed-loop MRgFUS, potentially combined with exposure fractionation, could overcome the challenges to chemotherapy delivery in glioblastomas.

Following the safety assessment of the proposed closed-loop MRgFUS system we evaluated the ability of localized thermal stress to trigger the release and enhance the delivery of Doxorubicin by TSL-Dox in brain tumors. Our investigations indicated a 3.5-fold and 5-fold improvement in Dox delivery in the GL261 glioma mice tumor and F98 glioma rat tumor models, respectively (Figures 3 and 4). Interestingly, this level is comparable to the (free) Dox delivery observed in the GL261 model using microbubble enhanced FUS (~ 4-fold) 47. Although these findings are encouraging, the quantification of Dox delivery using fluorescence microscopy or fluorimetry, with doxorubicin being used as the fluorophore, can be impacted by pH, protein binding, drug crystallization, and whether the drug is still encapsulated (where it may crystallize or self-quench) 51. Hence, these data, in terms of absolute values, should be interpreted with some caution. Employing more sensitive and quantitative methods to assess the concentration of Dox and its metabolites (e.g. mass spectrometry) will allow to more accurately quantify the amount of Dox delivered and its distribution 52.

The observed improvement in Dox delivery is also comparable with preclinical work in extracranial malignancies (on average 7.8-fold improvement in Dox delivery as compared to control, TSL-Dox only; Table S1), where the tumor vessels are both substantially leakier and their pore size can increase substantially in response to thermal stress (> 4-fold) 53,54. Based on these observations we formed the hypothesis that thermal stress may trigger changes in the permeability of the brain vessels. Mathematical inference and prospective experimental investigations (Figures 5 and 6) supported our hypothesis and revealed that thermal stress, indeed, changes the cerebrovascular transport dynamics in gliomas. While the inferred parameters (e.g. BBB diffusion coefficient) cannot be directly correlated to the measured K^trans^ values, which reflect the combined effects of blood flow, vascular permeability, and capillary surface area 55, these prospective measurements indicated that the model predictions were in the right direction. Additional investigations to elucidate further the role of thermal stress in the cerebrovascular transport dynamics in the brain tumor microenvironment are warranted.

Our investigations indicated that these changes in the transport dynamics could also improve the delivery of (un-encapsulated) small molecule chemotherapeutics, such as Dox. Crucially, this trend was apparent only when Dox was administered during the application of thermal stress, suggesting that the observed effects are transient. It is possible that the cell uptake is also enhanced during the application of FUS-hyperthermia, our *in vitro* investigations suggest that it affects the rate of Dox uptake, which for drugs that are cleared fast, such as, doxorubicin, this can be critical (Figure S4B). Taken together our investigations not only highlighted the transient nature of the observed effect, but also revealed the ability of FUS-mediated thermal stress to establish and refine tumor-specific treatment windows (spatial and temporal) to increase the drug delivery efficacy.

While collectively our data support the hypothesis that US thermal stress increases the vessel permeability, additional effects might have contributed to our observations 56. Perhaps we could exclude US-mediated changes in IFP, as IFP in brain tumors is not considered to be as high as it is in extracranial malignancies 57. Likewise, convective transport is very slow compared to diffusive transport for small molecules (i.e., the locally released Dox is likely not bound to albumin), potentially diminishing its contribution to the observed changes in the drug delivery. This is further supported by our numerical investigations that showed small changes in hydraulic conductivity after the application of thermal stress (Figure 5B). Thus, it is more likely that changes in vascular and transvascular transport are the main biological factors by which heat increases drug delivery in brain tumors. Nevertheless, additional investigations with larger molecules, direct measurement of IFP, and reduced parametric uncertainty in the system identification procedures (i.e., estimation of smaller number of transport parameters) should be considered to further support the above reasoning and findings. These investigations may also lead to improved drug delivery protocols.

In our investigations, we were also not able to decouple the effects of thermal with mechanical stress, which inevitably was present. Therefore, mechanical stress induced by the sonications might also have caused additional changes in the brain tumor microenvironment that might have influenced the observed transport and drug uptake patterns 58. For instance, recent *ex vivo* analysis of brain tissue indicated that US pulses designed to induce mechanical stress could expand the extracellular and perivascular spaces 59, thereby reducing the resistance to flow. Although it is not clear to what extent the observed increase in drug accumulation is caused by mechanical stress (US), thermal stress, and triggered drug release, our investigations provided evidence that non-ablative FUS can transiently modify the brain tumor microenvironment and its transport dynamics. Thereby, creating new opportunities for targeted drug delivery in aggressive brain tumors, such as glioblastomas.

Finally, our findings attained comparable improvement in survival with microbubble enhanced FUS combined with free Dox. The fact that this drug delivery strategy (i.e., microbubble enhanced FUS) is currently under clinical evaluation against glioblastoma with promising early findings 60, highlights the potential of the proposed strategy and its ability to implement intensified chemotherapy protocols. Crucially, the proposed strategy due to encapsulation can potentially lead to much lower systemic toxicity. It is also conceivable that the therapeutic efficacy of the proposed therapeutic strategy can be further improved using exposure fractionation, as we alluded to above. In addition to refining the experimental conditions, combining the employed thermosensitive drug delivery technology with other chemotherapeutic agents, such as cisplatin 61 or carboplatin, may allow to further improve outcomes. Likewise, more detailed survival analysis, using different tumor models (e.g., glioblastoma xenografts, breast brain metastasis, etc.) and additional control groups (e.g., no drug, free Dox, FUS hyperthermia alone, etc.) is needed to fully evaluate the therapeutic potential of the proposed strategy.

Besides employing different strategies to improve treatment outcomes, safety will remain an important consideration for translating the proposed strategy to the clinics, especially as new protocols are evaluated. While our findings suggest that the proposed treatment has minimal impact on the brain (Figure 2), a more detailed and quantitative assessment of the BBB phenotype (i.e., structure and function) and brain tissue 62,63 at different FUS exposures (e.g. duration) and time points will allow to further define and refine the treatment window for safe and effective drug delivery in the brain tumor microenvironment. Moreover, direct assessment of the brain function and response to mild hyperthermia (e.g., electrophysiological monitoring) will allow to better characterize and understand its (transient) response to thermal stress as well as assesses potential adverse effects. Although, in healthy brain the BBB is disrupted only after sub-ablative thermal doses have been reached 64,65, it will be interesting to assess the relationship between BBB permeability and thermal stress at the tumor margin, where infiltrating cancer resides and currently remain inaccessible to therapy 12. Finally, assessing potential off target effects of the released drug will allow to better characterize the safety profile of the proposed strategy and facilitate its translation to the clinic.

## Conclusion

We have successfully demonstrated that closed-loop MRgFUS mild hyperthermia in combination with TSL-Dox can significantly enhance the delivery of chemotherapy into glioma tumors. Moreover, we characterized the impact of thermal stress on cerebrovascular transport dynamics via DCE-MRI and mathematical modeling and found that thermal stress can trigger acute changes in the vascular transport dynamics in glioma tumors. Together our findings not only suggest that localized transcranial MRgFUS-hyperthermia can increase Dox accumulation in brain tumors by TSL-Dox, but also support the hypothesis that this enhanced delivery is due to combined effects of FUS-triggered drug release and thermal stress-mediated changes in key transport parameters. Overall, our findings suggest that the proposed targeted drug release and delivery approach can potentially lead to a clinically viable treatment strategy against glioblastomas.

## Supplementary Material

Supplementary figures and tables.

## Figures and Tables

**Table 1:** Variables and values used in the Bio-heat equation (taken from 33)

Parameters	Description	Value
*k*	Thermal conductivity of tissue	0.6 W/(m⋅K)
*ρ*	Density of tissue	1000 kg/m^3^
*c*	Specific heat of tissue	3500 J/(kg⋅K)
*ρ_bl_*	Density of blood	1060 kg/m^3^
*c_bl_*	Specific heat of blood	3800 J/(kg⋅K)
*w_bl_*	Blood perfusion rate	0.018 1/s
*τ_bl_*	Arterial blood temperature	37 °C

**Table 2:** Defined Parameters for PBPK model

Parameters	Description
*D_v_*	Vessel effective diffusion coefficient
*Di*	Interstitium diffusion coefficient
*K*	Interstitium hydraulic conductivity
*V*	Rate of transmembrane transport
*V_b_*	Rate of drug binds to cellular DNA

**Table 3:** Inferred transport parameters of the GL261 tumors with (FUS) and without (Non-FUS) the application of localized thermal stress. The values are plotted in Figure 5B

Parameters	Description	Non-FUS	FUS	Unit	P Value
*Dv*	Vessel effective diffusion coefficient	5.2x10^-13^ ± 2.0x10^-14^	1.2x10^-12^ ± 3.5x10^-12^	μm^2^/s	0.00074
*Di*	Interstitium diffusion coefficient	4.2x10^-11^ ± 1.9x10^-12^	4.0x10^-11^ ± 5.5x10^-13^	μm^2^/s	0.2306
*K*	Interstitium hydraulic conductivity	3.2x10^-14^ ± 8.6x10^-16^	3.0x10^-14^ ± 1x10^-16^	m^3^∙s/kg	0.0788
*V*	Rate of transmembrane transport	4.0x10^-5^ ± 2.0x10^-6^	4.4x10^-5^ ± 7.8x10^-7^	nM/s	0.1108
*Vb*	Rate of drug binds to cellular DNA	1.1x10^-4^ ± 1.0x10^-5^	1.5x10^-3^ ± 6.4x10^-4^	1/s	0.1047

## Abbreviations

FUS: Focused Ultrasound
Dox: Doxorubicin
TSL: Thermosensitive Liposomes
PBPK: Physiologically Based Pharmacokinetic Modeling
MRgFUS: Magnetic Resonance guided Focused Ultrasound
