# Supplementary material for: Closed-loop trans-skull ultrasound hyperthermia leads to improved drug delivery from thermosensitive drugs and promotes changes in vascular transport dynamics in brain tumors
Source: Theranostics. 2021 May 24;11(15):7276–93. doi: 10.7150/thno.54630 (PMC8210606; doi:10.7150/thno.54630)
Supplement: Supplementary file 1 — Supplementary figures and tables. [file thnov11p7276s1.pdf]

Supplementary material

Closed-loop trans-skull ultrasound hyperthermia leads to improved drug delivery from thermosensitive drugs and promotes changes in vascular transport dynamics in brain tumors

Chulyong Kim[1], Yutong Guo[1], Anastasia Velalopoulou[1,2], Johannes Leisen[3], Anjan Motamarry[4], Krishna Ramajayam[5], Muna Aryal[6], Dieter Haemmerich[5], and Costas D. Arvanitis[1,2*]

[1]School of Mechanical Engineering, Georgia Institute of Technology, Atlanta, USA

[2]Department of Biomedical Engineering, Georgia Institute of Technology and Emory University, Atlanta, GA, USA

[3]Department of Chemistry and Biochemistry, Georgia Institute of Technology, Atlanta, GA, USA

[4] Department of Dermatology, Massachusetts General Hospital, Harvard Medical School, Boston, MA, USA

[5]Department of Pediatrics, Medical University of South Carolina, Charleston, SC, USA

[6]Department of Radiology, Stanford University, Stanford, CA, USA

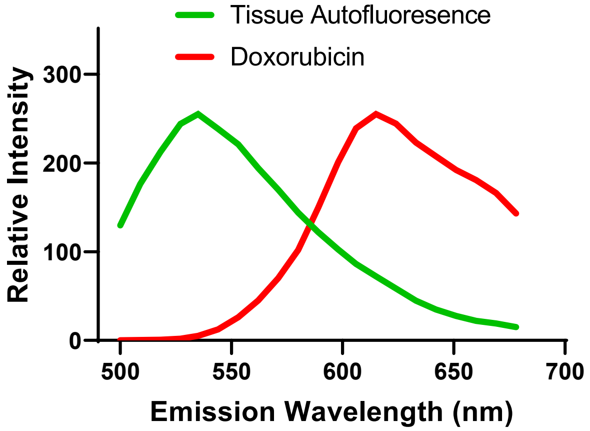

Figure S1. Spectral imaging emission wavelengths of tissue autofluorescence and Doxorubicin.

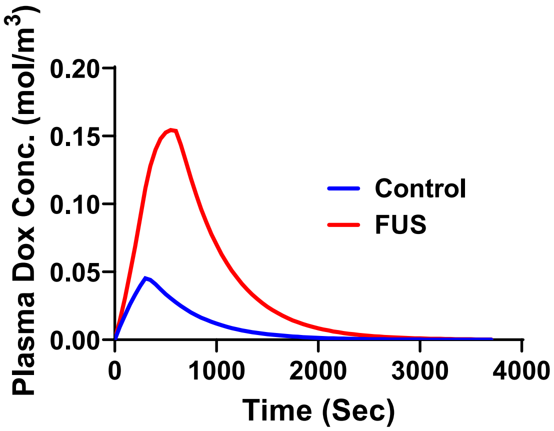

Figure S2. Calculated plasma Dox concentration for numerical modeling

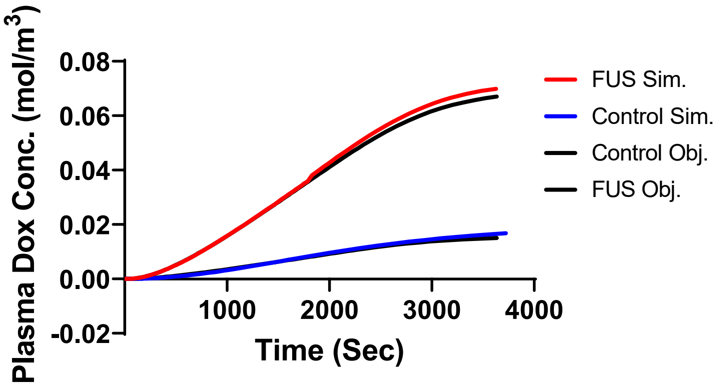

Figure S3. Objective input functions vs. optimization fits.

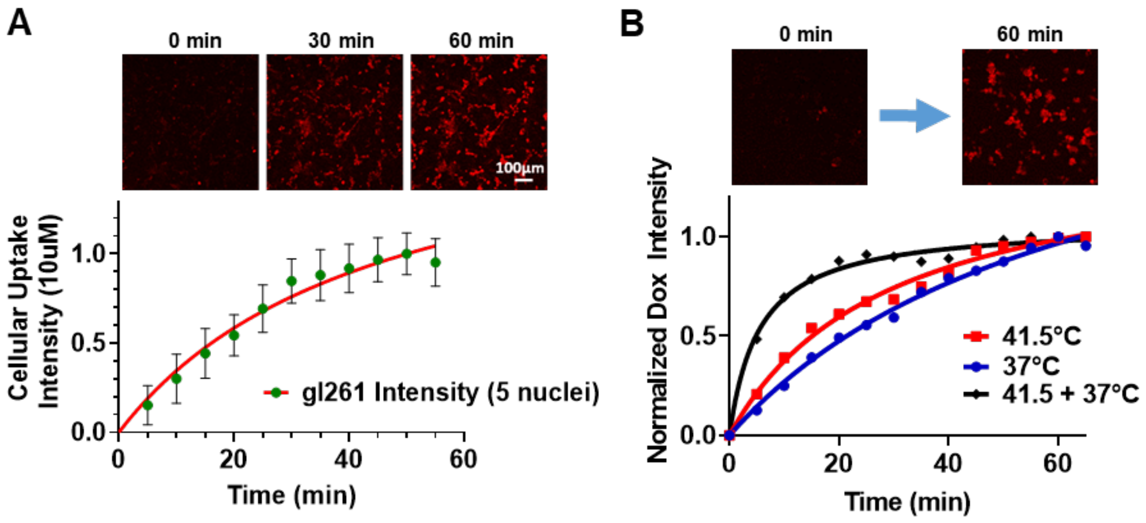

Figure S4. In-vitro characterization of Dox release from TSL and uptake from GL216 cells. (A) Transient drug uptake behaviors of gl261 cells from 0 to 60 min. (B) Transient drug uptake behavior of gl261 in different temperature exposure conditions.

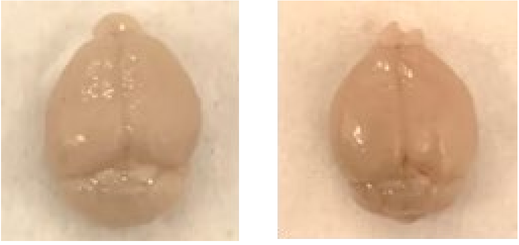

Figure S5. Images of harvested brain tissues (target temperature was 41.5°C for 10 mins) indicating minimal tissue damage at the surface of the brain.

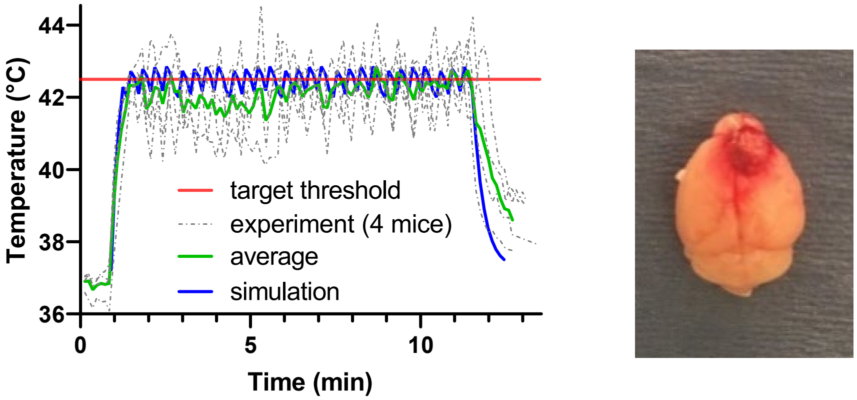

Figure S6. *Left:* Temperature profiles of FUS experiment at 42.5^{0}C. *Right:* Harvested brain indicating extended hemorrhage.

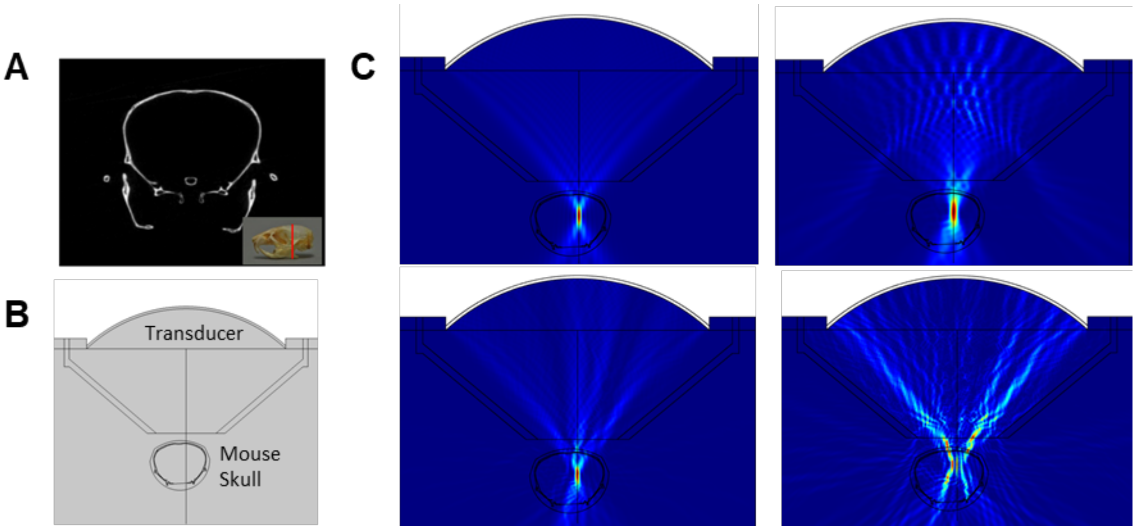

Figure S7. (A) A mouse skull was CT-scanned with 50 μm voxel size. (B) CT image was incorporated into Comsol Multiphysics to create numerical model. The frequency range from 1 MHz to 2 MHz were simulated and (C) Acoustic simulation revealed to have optimized frequency around 1.7 MHz (free field – top left vs. skull incorporated – bottom left (1.7 MHz). (1 MHz – top right, 2 MHz – bottom right)

Table S1. Review on main publications on HSL-Dox. These data were used to extract mean values on dox delivery and improvement in survival.

Tumor	Drug Type / Dosage	Controller (Y/N)	Exposure	Measurement	Main Finding (fold increase / survival)	Open Question	Ref
				**FUS**			
Mice / Human FaDu squamous cell carcinoma cells	HSL-Dox 10 mg/kg	Yes	FUS ~42°C, 10 x 30s bursts	Intravital two photon microscopy (2PM)	10 x 30s bursts show about half of interstitial drug concentration from 20min continuous sonication (~6 fold longer exposure time). 26.7-fold drug delivery enhancement in animal tumor.	The study does not quantify the effect of local hyperthermia in tumor environment.	[1]
Rat / Rhabdomyosarcoma rat tumor	HSL-Dox / 5mg/kg (exp), 2mg/kg (survival)	MR thermometry monitoring	FUS ~41°C, 2 x 15min	Single-photon emission CT (SPECT) / Fluorescence microscopy	For biodistribution of dox 90 min after injection of the TSL more dox was delivered in the tumors. Control vs. hyperthermia resulted in 3.7-fold increase (~1 vs. ~3.7 % ID/g). Heat + TSL treatment led to a decrease in tumor growth.	Lack of quantification on cellular uptake and of the effect of local hyperthermia in tumor environment.	[2]
Rat / 13762 MAT B III cells injected in the hind lib of rats	NTSL, HSL, PTSL (sensitive to temp + pH)	Yes	FUS ~40°C / 43°C, 5min	Microscopy	Admin. of PTSL+FUS led to the greatest reduction in tumor growth.	The biodistribution of the drugs and the effect of local hyperthermia in tumor environment was not quantified.	[3]
Rabbit / Thigh muscle	HSL-Dox, 5mg/kg	Yes	FUS ~42°C, 30min	Fluorescence microscopy	~6 times higher Dox fluorescence averaged over a 5 mm diameter.	Detailed drug distribution and the effect of local hyperthermia in tumor environment was not shown.	[4]

Mice / Murine breast cancer	Pegylated liposomal Dox (PLD) / 5 mg/kg	No	FUS ~42-43°C, 10min	Fluometry	Dox concentration in tumor for PLD + FUS was 2.4-fold that of the PLD only group. PLD + hyperthermia markedly inhibited breast tumor growth in the brain.	Drug and temp distribution along with and the effect of local hyperthermia in tumor environment was not quantified.	[5]
Rabbits / Thigh	HSL-Dox / 2.5 mg/kg	Yes	FUS ~43°C, 20-30min	Fluometry (homogenized tissue)	Drug concentrations in heated regions were, on average 15.8 times (8.3 vs. 0.5 ng/mg) higher than in the corresponding unheated regions of the contralateral thigh.	Effects of temperature, heating duration, and thermal dose on drug deposition not shown.	[6]
**Different heating methods**							
Mice / Human tumor (SKOV-3 ovarian carcinoma)	No therapeutic agent / Rhodamine labeled liposomes	No	HT chamber (fluid)	Intravital microscopy	For ~1h treatment the relative concentration of liposomes in the tumor were ~0, 0.46, 0.94, 1.53 @ 34-39°C, 40°C, 41°C, 42°C	Unknown if these observations apply to brain tumors	[7]
Rats / Healthy	Albumin – positive cells assessed	No	Pentobarbital by inhibiting brain metabolism	Immunostaining	@ temp (34.2-38.0°C), (34.2-42.3°C), (38.0-42.5°C), albumin-positive cells increase to (1.90+/-0.74 vs. 8.50+/-2.22 vs. 17.71+/-2.67)	Unknown if these observations apply to brain tumors	[8]
Canines / Healthy Brain	HSL-Dox/ 0.94 mg/kg	No	RF generator	high-performance liquid chromatography, fluorescence microscopy	Dox delivery ranged from 0.11 to 0.74 ug/g of brain tissue at the hyperthermia locations, with undetectable drug uptake in unheated tissue. No animals in the survival group demonstrated significant neurological deficits.	Unknown if these observations apply to brain tumors	[9]

Mice / human squamouse cell carcinoma	HSL-Dox/ 5 mg/kg	No	Water bath (leg)		Growth time (x5 of initial tumor size) = 19.8 (control – saline) vs. 51.4 (LTSL @ 42°C)	Drug biodistribution and uptake not quantified	[10]
Rodent / Subcutaneous rat R1 rhabdomyosarcoma	HSL-Dox / 4 mg/kg	No	Water Bath	Fibered confocal fluorescence microscopy	Reported cell uptake rates (1/k) of 3 minutes (n = 241 cells) and a released-DOX penetration in the range of 2500 µm2·s−1 in the tumor extravascular space.	Unknown if these observations apply to brain tumors.	[11]
Mice / Pancreatic subcutaneous tumors	GNRs (stealth gold nanorods)	No	Pulsed laser irradiation	Evans blue dye / fluorescent dextran dye	Blood perfusion increases with rising temperature and peaks at a 11-fold increase relative to baseline. The treatment increased vascular permeability within 24 h after treatment, allowing enhanced transport of macromolecules up to 54 nm in size. Dye extravasation over the unheated control (2-fold increase at 1 h and 2.5-fold increase at 3 h)	Unknown if these observations apply to brain tumors.	[12]
Mice / Murine B16 melanoma, BFS-1 sarcoma, Lewis Lung Carcinoma, Human BLM melanoma	HSL-Dox/ 6 µmol lipid	No	Heating coil attached to dorsal window chamber	Intravital fluorescence microscopy	Liposomes intensity increased in the EES over time up to 1h at 41°C in all tumor models. Increased exposure caused increased extravasation. The extravasation is noticed in EES up to 27.5 µm from the vessel. Increased permeability is effective for up to 8 hr.	Unknown if these observations apply to brain tumors.	[13]

Mice / Murine BFS-1 sarcoma, human BLM melanoma	HSL-Dox/ 7mg/kg	No	Heating coil attached to dorsal window chamber	Intravital fluorescence microscopy	TSL + HT (42°C) showed intravascular Dox release with peak Dox concentration (penetration) observed at 50 min of heating. Dox intensity increased to more than 8-fold compared to initial during heating period of 40 min. TSL + HT had tumor growth delay of ~16 days.	Unknown if these observations apply to brain tumors	[14]
Mice / subcutaneous tumors	HSL-Dox / 5mg/kg	No	Custom HT probe (thermistor)	In-vivo fluorescence imaging / Liquid chromatography	Drug related fluorescence of heated tumors (~43°C) increased by 4.6-fold (15min HT), 9.3-fold (30min HT), and 13.2-fold (60min HT). Tumor drug concentrations were 4.2 ug/g (no HT), 7.1 ug/g (15min), 14.1 ug/g (30min HT), 21.4 ug/g (60min HT).	What is the cellular uptake of the drug? Unknown if these observations apply to brain tumors.	[15]

Table S2: PBPK Model Parameters

Symbol	Description	Value	Reference
V_{rbc}	RBC velocity in tumor vessels	0.12 mm/s	[16]
PV	Vascular pressure	5 mmHg	[17]
PI	Interstitial pressure	1 mmHg	-
μ	Blood viscocity	0.004 Pa*s	[17],[18],[19]
εi	Interstitium porosity	0.4	[19–22]
εv	Vessel wall porosity	0.5	-
K	Hydraulic conductivity	$4x10^{-8}$ cm^2/(mmHg*s)	[23]
d	Vessel wall thickness	5 µm	[17]
D_v	Vessel effective diffusion coefficient	1.25 μm^2/sec	[18,24]
D_i	Interstitium effective diffusion coefficient (free dox)	40 μm^2/sec	[18,19,25]
D_b	Diffusion coefficient in blood	$1x10^5$ μm^2/sec	[26]
V	Rate of transmembrane transport	42.9 nM/s	[18,19,27]
V_b	Rate of drug binding to nucleus	0.0016 1/s	
K_e	Michaelis-Menten kinetics constants	403 nM	
K_i		63 µM	
BW	Body weight for mice	20 g	Assumed
Vtot	Total blood volume in body	1.17 ml	
D	Total dose of encapsulated Dox injected	0.14mg	Calculated
Hct	Hematocrit	0.45	[28]
V_p^B	Volume of systemic plasma	-	Vtot*(1-Hct)
Ke_dox	Dox clearance constant	2.1e-3 s^{-1}	[29]
Ke_hsl	Rate constant of HSL clearance	2.228e-4 s^{-1}	
R_37	Release rate of Dox from HSL at 37°C	Variable s^{-1}	Calculated
R_41.5	Release rate of Dox from HSL at 41.5°C	Variable s^{-1}	Calculated
CBF_perf	Brain CBF perfusion rate	1.07 ml/g/min	[30]

References

1. Santos MA, Goertz DE, Hynynen K. Focused Ultrasound Hyperthermia Mediated Drug Delivery Using Thermosensitive Liposomes and Visualized With *in vivo* Two-Photon Microscopy. Theranostics. 2017; 7: 2718–31.
2. Hijnen N, Kneepkens E, de Smet M, Langereis S, Heijman E, Grüll H. Thermal combination therapies for local drug delivery by magnetic resonance-guided high-intensity focused ultrasound. Proc Natl Acad Sci. 2017; 114: E4802–11.
3. Ta T, Bartolak-Suki E, Park E-J, Karrobi K, McDannold NJ, Porter TM. Localized delivery of doxorubicin in vivo from polymer-modified thermosensitive liposomes with MR-guided focused ultrasound-mediated heating. J Controlled Release. 2014; 194: 71–81.
4. Gasselhuber A, Dreher MR, Partanen A, et al. Targeted drug delivery by high intensity focused ultrasound mediated hyperthermia combined with temperature-sensitive liposomes: Computational modelling and preliminary *in vivo* validation. Int J Hyperthermia. 2012; 28: 337–48.
5. Wu S-K, Chiang C-F, Hsu Y-H, et al. Short-time focused ultrasound hyperthermia enhances liposomal doxorubicin delivery and antitumor efficacy for brain metastasis of breast cancer. Int J Nanomedicine. 2014; 9: 4485–94.
6. Staruch R, Chopra R, Hynynen K. Localised drug release using MRI-controlled focused ultrasound hyperthermia. Int J Hyperthermia. 2011; 27: 156–71.
7. Kong G, Braun RD, Dewhirst MW. Characterization of the Effect of Hyperthermia on Nanoparticle Extravasation from Tumor Vasculature. Cancer Res. 2001; 61: 3027–32.
8. Kiyatkin EA, Sharma HS. Permeability of the blood-brain barrier depends on brain temperature. Neuroscience. 2009; 161: 926–39.
9. Bredlau AL, Motamarry A, Chen C, et al. Localized delivery of therapeutic doxorubicin dose across the canine blood–brain barrier with hyperthermia and temperature sensitive liposomes. Drug Deliv. 2018; 25: 973–84.
10. Anyarambhatla GR, Needham D. Enhancement of the Phase Transition Permeability of DPPC Liposomes by Incorporation of MPPC: A New Temperature-Sensitive Liposome for use with Mild Hyperthermia. J Liposome Res. 1999; 9: 491–506.
11. Derieppe M, Escoffre J-M, Denis de Senneville B, et al. Assessment of Intratumoral Doxorubicin Penetration after Mild Hyperthermia-Mediated Release from Thermosensitive Liposomes. Contrast Media Mol Imaging. 2019; 2019: 1–13.
12. Kirui DK, Koay EJ, Guo X, Cristini V, Shen H, Ferrari M. Tumor vascular permeabilization using localized mild hyperthermia to improve macromolecule transport. Nanomedicine Nanotechnol Biol Med. 2014; 10: 1487–96.
13. Li L, ten Hagen TLM, Bolkestein M, et al. Improved intratumoral nanoparticle extravasation and penetration by mild hyperthermia. J Controlled Release. 2013; 167: 130–7.
14. Li L, ten Hagen TLM, Hossann M, et al. Mild hyperthermia triggered doxorubicin release from optimized stealth thermosensitive liposomes improves intratumoral drug delivery and efficacy. J Controlled Release. 2013; 168: 142–50.

15. Motamarry A, Negussie AH, Rossmann C, et al. Real-time fluorescence imaging for visualization and drug uptake prediction during drug delivery by thermosensitive liposomes. Int J Hyperthermia. 2019; 36: 817–26.

16. Kamoun WS, Chae S-S, Lacorre DA, et al. Simultaneous measurement of RBC velocity, flux, hematocrit and shear rate in vascular networks. Nat Methods. 2010; 7: 655–60.

17. Stylianopoulos T, Jain RK. Combining two strategies to improve perfusion and drug delivery in solid tumors. Proc Natl Acad Sci. 2013; 110: 18632–7.

18. Thurber GM, Weissleder R. A Systems Approach for Tumor Pharmacokinetics. Boswell CA, Ed. PLoS ONE. 2011; 6: e24696.

19. El-Kareh AW, Secomb TW. A Mathematical Model for Comparison of Bolus Injection, Continuous Infusion, and Liposomal Delivery of Doxorubicin to Tumor Cells. Neoplasia. 2000; 2: 325–38.

20. Panagiotaki E, Walker-Samuel S, Siow B, et al. Noninvasive Quantification of Solid Tumor Microstructure Using VERDICT MRI. Cancer Res. 2014; 74: 1902–12.

21. Vincensini D, Dedieu V, Eliat PA, et al. Magnetic resonance imaging measurements of vascular permeability and extracellular volume fraction of breast tumors by dynamic Gd-DTPA-enhanced relaxometry. Magn Reson Imaging. 2007; 25: 293–302.

22. Miller MA, Gadde S, Pfirschke C, et al. Predicting therapeutic nanomedicine efficacy using a companion magnetic resonance imaging nanoparticle. Sci Transl Med. 2015; 7: 314ra183-314ra183.

23. Swabb EA, Wei J, Cullino PM. Diffusion and Convection in Normal and Neoplastic Tissues. 1974; 34: 10.

24. Wu NZ, Klitzman B, Rosner G, Needham D, Dewhirst MW. Measurement of material extravasation in microvascular networks using fluorescence video-microscopy. Microvasc Res. 1993; 46: 231–53.

25. Weinberg BD, Patel RB, Exner AA, Saidel GM, Gao J. Modeling doxorubicin transport to improve intratumoral drug delivery to RF ablated tumors. J Controlled Release. 2007; 124: 11–9.

26. Pluen A, Boucher Y, Ramanujan S, et al. Role of tumor-host interactions in interstitial diffusion of macromolecules: Cranial vs. subcutaneous tumors. Proc Natl Acad Sci. 2001; 98: 4628–33.

27. Jackson TL. Intracellular Accumulation and Mechanism of Action of Doxorubicin in a Spatio-temporal Tumor Model. J Theor Biol. 2003; 220: 201–13.

28. Tofts PS, Brix G, Buckley DL, et al. Estimating kinetic parameters from dynamic contrast-enhanced t1-weighted MRI of a diffusable tracer: Standardized quantities and symbols. : 10.

29. Gasselhuber A, Dreher MR, Rattay F, Wood BJ, Haemmerich D. Comparison of Conventional Chemotherapy, Stealth Liposomes and Temperature-Sensitive Liposomes in a Mathematical Model. Urtti A, Ed. PLoS ONE. 2012; 7: e47453.

30. Muir ER, Shen Q, Duong TQ. Cerebral blood flow MRI in mice using the cardiac-spin-labeling technique. Magn Reson Med. 2008; 60: 744–8.
